# The transcription factor BCL11A restores differentiation potential to aged oligodendrocyte progenitor cells

**DOI:** 10.7554/eLife.110038

**Published:** 2026-10-26

**Authors:** Tanay Ghosh, Roey Baror, Chao Zhao, Amar Sharma, Wing Hei Au, András Lakatos, Nick Goldman, Robin JM Franklin

**Affiliations:** 1 Altos Labs-Cambridge Institute of Science Cambridge United Kingdom; 2 Wellcome-MRC Cambridge Stem Cell Institute, University of Cambridge Cambridge United Kingdom; 3 Department of Clinical Neurosciences, University of Cambridge Cambridge United Kingdom; 4 John Van Geest Centre for Brain Repair, University of Cambridge Cambridge United Kingdom; 5 European Molecular Biology Laboratory, European Bioinformatics Institute, Wellcome Genome Campus Hinxton United Kingdom; University of California, San Francisco United States; University of Hong Kong Hong Kong

**Keywords:** ageing, gSWITCH, oligodendrocyte progenitor cells, OPCs, time series, Mouse, Rat

## Abstract

In young animals, oligodendrocyte progenitor cells (OPCs) in the CNS efficiently differentiate into myelinating oligodendrocytes (OLs), whereas ageing reduces this capacity, impairing remyelination following demyelinating injury and adaptive myelination involved in learning. To identify factors that restore resilience in aged OPCs, we developed gSWITCH (https://altoslabs.shinyapps.io/gSWITCH/, https://github.com/Tanay-math/gSWITCH), a computational pipeline that captures dynamic gene-expression patterns to identify ‘switch’ genes during lineage progression and ordered biological transitions, including time-series experiments. gSWITCH identified OPC-characteristic switch genes, which we compared between young and aged OPCs to determine whether their regulation is altered with ageing. This revealed several transcription factors with reduced expression in aged OPCs. Transcription-factor binding-site enrichment highlighted *Bcl11a* as a potential master-regulator of multiple switch genes. *Bcl11a* inhibition reduced OPC differentiation into OLs, whereas its overexpression in aged OPCs significantly enhanced OL differentiation without affecting proliferation. In aged mice subjected to demyelination injury in spinal cord white matter, *Bcl11a* overexpression increased OPC differentiation within lesions. We found that *Bcl11a* has undergone pervasive purifying selection throughout vertebrate evolution, preserving its amino acid sequence by eliminating deleterious mutations. Our study shows that reversing the age-related decline of this evolutionarily conserved factor in aged OPCs restores their impaired capacity for differentiation.

## Introduction

The regulation of gene expression during development is highly dynamic, guiding the transition from progenitor cells to terminally differentiated cell types (Peter and Davidson, 2011; Pope and Medzhitov, 2018). This temporal control ensures that appropriate gene expression programs are activated at each stage of lineage progression. Mapping these regulatory programs and identifying key drivers within gene networks is essential for understanding how cell fate decisions are made and maintained. However, this developmental plasticity reduces with age, as progenitor cells progressively lose their ability to differentiate, impairing tissue homeostasis and repair (Rando et al., 2025; Brunet et al., 2023).

In the central nervous system (CNS), oligodendrocytes (OLs) are responsible for producing myelin, the lipid-rich sheath that insulates axons and accelerates electrical impulse conduction (Nave and Werner, 2014). In young animals, oligodendrocyte progenitor cells (OPCs) exhibit strong differentiation potential, transitioning through defined intermediate stages to generate mature, myelinating OLs. With ageing, however, this capacity declines: OPCs in older individuals show reduced ability to complete lineage progression, leading to compromised myelin maintenance, poor remyelination following demyelination injury and impaired adaptive myelination as a mechanism of learning (Franklin and Ffrench-Constant, 2017, Sim et al., 2002). The molecular mechanisms underlying this age-related block in OPC differentiation remain poorly understood. Elucidating the regulatory factors that control OL lineage progression – and identifying their misregulation in aged OPCs – could uncover novel targets to restore resilience in these cells.

Here, we employed a data-driven approach to uncover novel factors essential for restoring resilience in aged OPCs. This involved the development of a computational pipeline, gSWITCH (accessible at: https://altoslabs.shinyapps.io/gSWITCH/, https://github.com/Tanay-math/gSWITCH, copy archived at Ghosh, 2026a), designed to capture precise dynamic gene expression patterns and identify potential ‘*switch*’ genes, including transcription factors, during lineage progression. Using this set of candidate switch genes, we performed a comparative analysis of gene expression between young and aged animals, which revealed a group of transcription factors downregulated in aged OPCs. Further analyses highlighted *Bcl11a* as a potential master regulator. *Bcl11a* expression was highest in OPCs and progressively decreased in Pre-OLs and OLs. Functional assays demonstrated that *Bcl11a* inhibition markedly reduced OPC differentiation into OLs. Overexpression of *Bcl11a* significantly enhanced differentiation in aged OPCs in vitro and following demyelination injury in the spinal cord of aged mice, without increasing OPC proliferation. *Bcl11a* is found only in vertebrates, and our molecular evolution analysis suggests that purifying selection has pervasively acted on this gene to preserve its amino acid sequences throughout vertebrate evolution. Our study showed that reversing the age-associated decline in expression of this evolutionarily conserved factor reinstates the differentiation capacity of aged OPCs.

## Results

### Identification of switch genes during OL lineage progression

**Figure 1.**
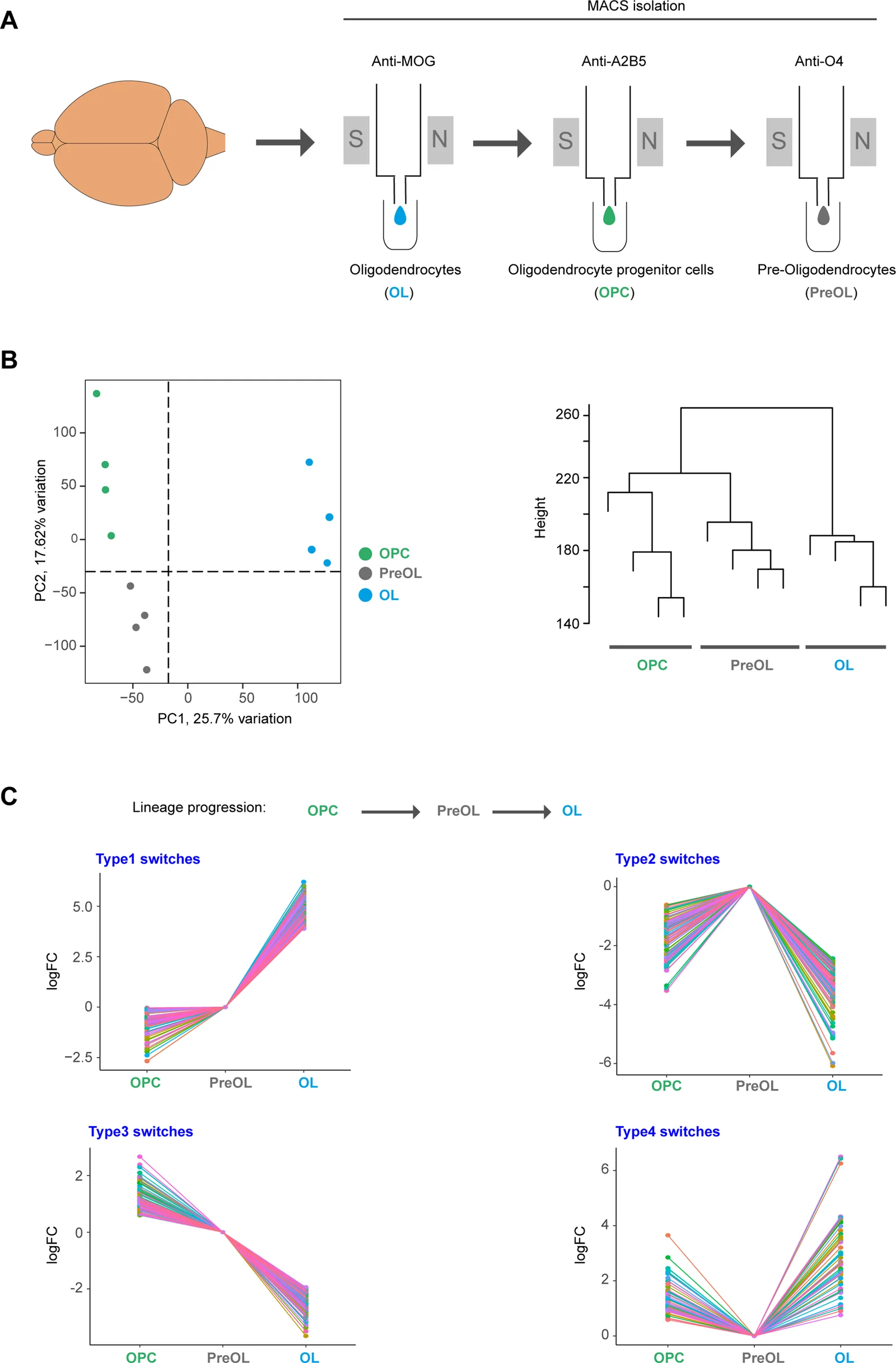
Dynamic regulation of genes during oligodendrocyte lineage progression. (**A**) Schematic diagram showing magnetic-activated cell sorting (MACS)-based isolation of oligodendrocyte progenitor cells (OPCs), pre-oligodendrocytes (PreOLs), and oligodendrocytes (OLs) from young rat brains (2–3 months). The schema illustrates the order in which these cell types were most effectively isolated: mature OLs were isolated first using the OL-specific marker MOG, followed by OPCs using A2B5, and finally PreOLs using O4. (**B**) Principal component analysis (PCA) (left) and average-linkage hierarchical clustering (right) based on global gene expression show that each cell type forms a distinct cluster. OPCs, PreOLs, and OLs were isolated from four different rats. (**C**) Using gSWITCH, we identified four distinct patterns of switch genes during OL lineage progression. Each coloured line represents one gene and connects its log_2_fold-change values across the three oligodendrocyte lineage states: OPC, PreOL, and OL. The connecting lines are used to visualise gene-wise patterns of expression change across these cell states. (**B, C**) GEO accession number of RNA-seq data: GSE301578. See also Supplementary file 1, Figure 1—figure supplement 1, Appendix 1, Figure 1—figure supplement 2, Figure 1—figure supplement 3A.

We performed RNA-seq analysis on OPC, Pre-OL, and OL isolated from young rat (2–3 months) brains (Figure 1A). The OPC, Pre-OL, and OL samples were clearly distinguishable based on their global gene expression profiles (Figure 1B). The expression patterns of cell-type-specific markers were consistent with their being distinct OPC, Pre-OL, and OL populations (Figure 1—figure supplement 1 see Appendix 1 for detailed description).

**Figure 1—figure supplement 1.**
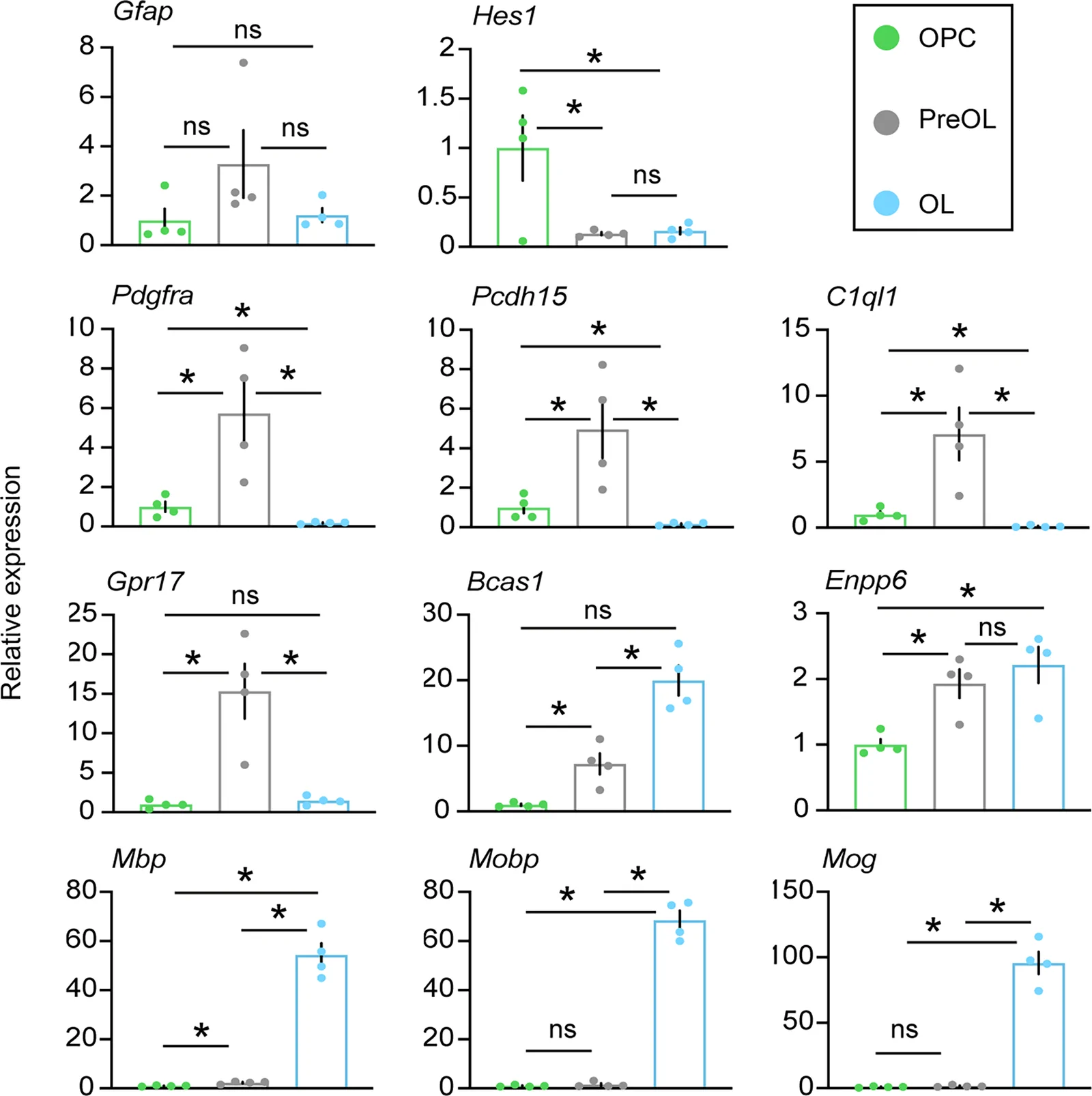
Normalised expression values of marker genes [for astrocytes, progenitor-state marker, oligodendrocyte progenitor cell (OPC), pre-oligodendrocytes (PreOL), and oligodendrocytes (OL)] from RNA-seq data (GEO accession number: GSE301578) were plotted relative to OPCs. n=4 (young rats), mean ± SEM, * adjusted p<0.05, Benjamini–Hochberg false discovery rate (FDR) after Wald test. ns: adjusted p>0.05. See also Appendix 1. Figure 1—figure supplement 1—source data 1.Numerical data underlying Figure 1—figure supplement 1.

**Figure 1—figure supplement 2.**
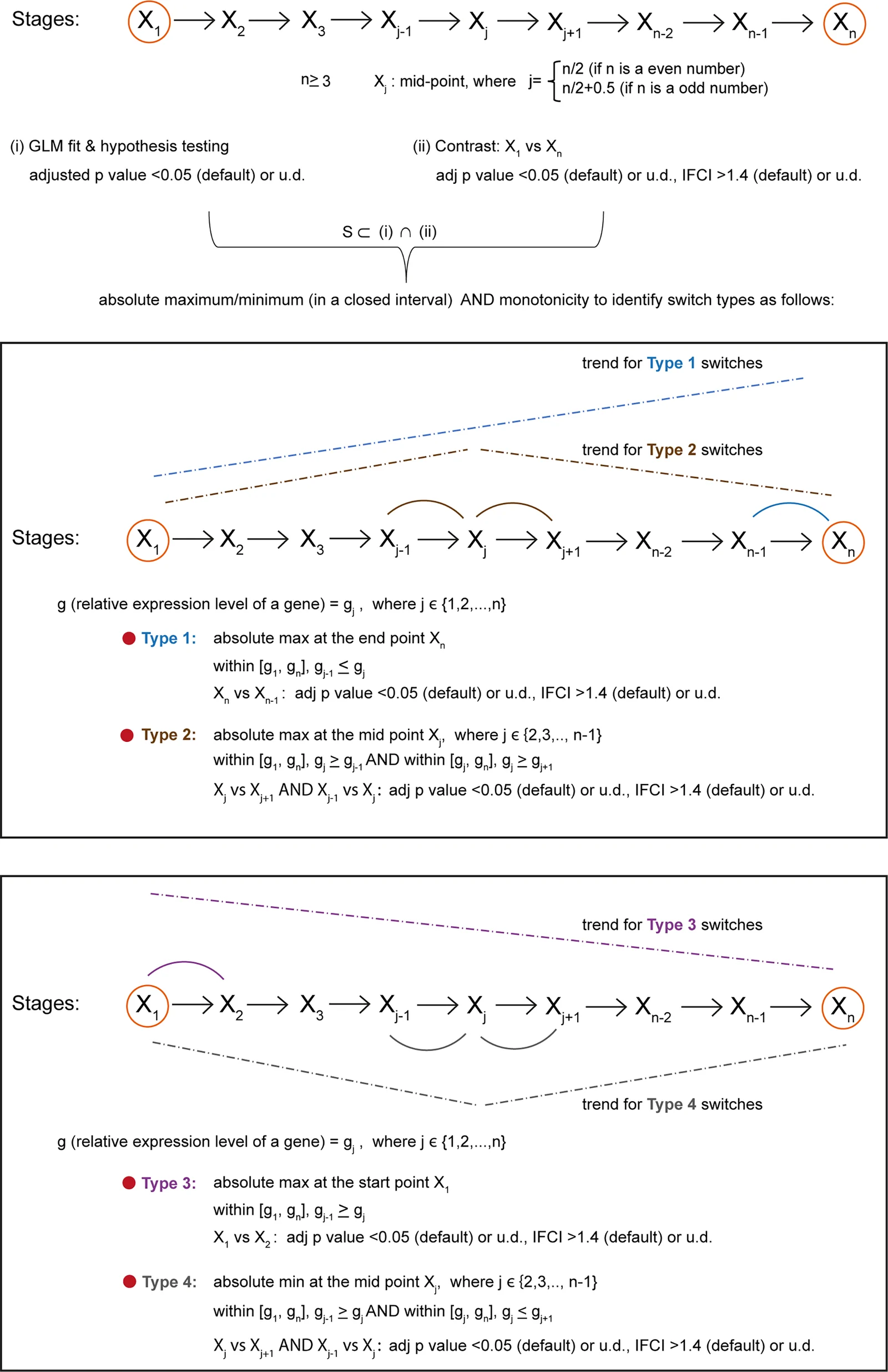
Schematic representation of the logic of the gSWITCH web application (for detail see ‘Materials and methods’ section). The gSWITCH application is accessible at https://altoslabs.shinyapps.io/gSWITCH/, https://github.com/Tanay-math/gSWITCH (Ghosh, 2026a). Red circles indicate the start and the end state of a series (time or lineage stages or any other variable-dependent series). GLM fit and hypothesis testing followed by contrast analysis between start and the end state generates a subset of genes on which we apply maximum (max) or minimum (min) criteria within a closed interval while taking into account of monotonicity trend, as well as further specific contrasts between groups. This allows identification of four types of switches. Detail criteria are shown inside the boxes and the trend pattern are represented by coloured dashed-straight lines. Arc or solid curve lines indicate statistical significance for specific contrast. Adj p-value: Adjusted p-value [Benjamini–Hochberg false discovery rate (FDR) after likelihood ratio test (LRT) or Benjamini–Hochberg FDR after Wald test for specific contrast analysis]. u.d.: user defined value. Note that to run gSWITCH, RNA-seq data are required (bulk or scRNA-seq flattened to pseudo bulk), where the number of series or time points or group >3 and the replicates per time point or group >2. gSWITCH also identifies transcription factors (TFs) within the switches and provides a summary of the total number of switches and TFs per switch type.

**Figure 1—figure supplement 3.**
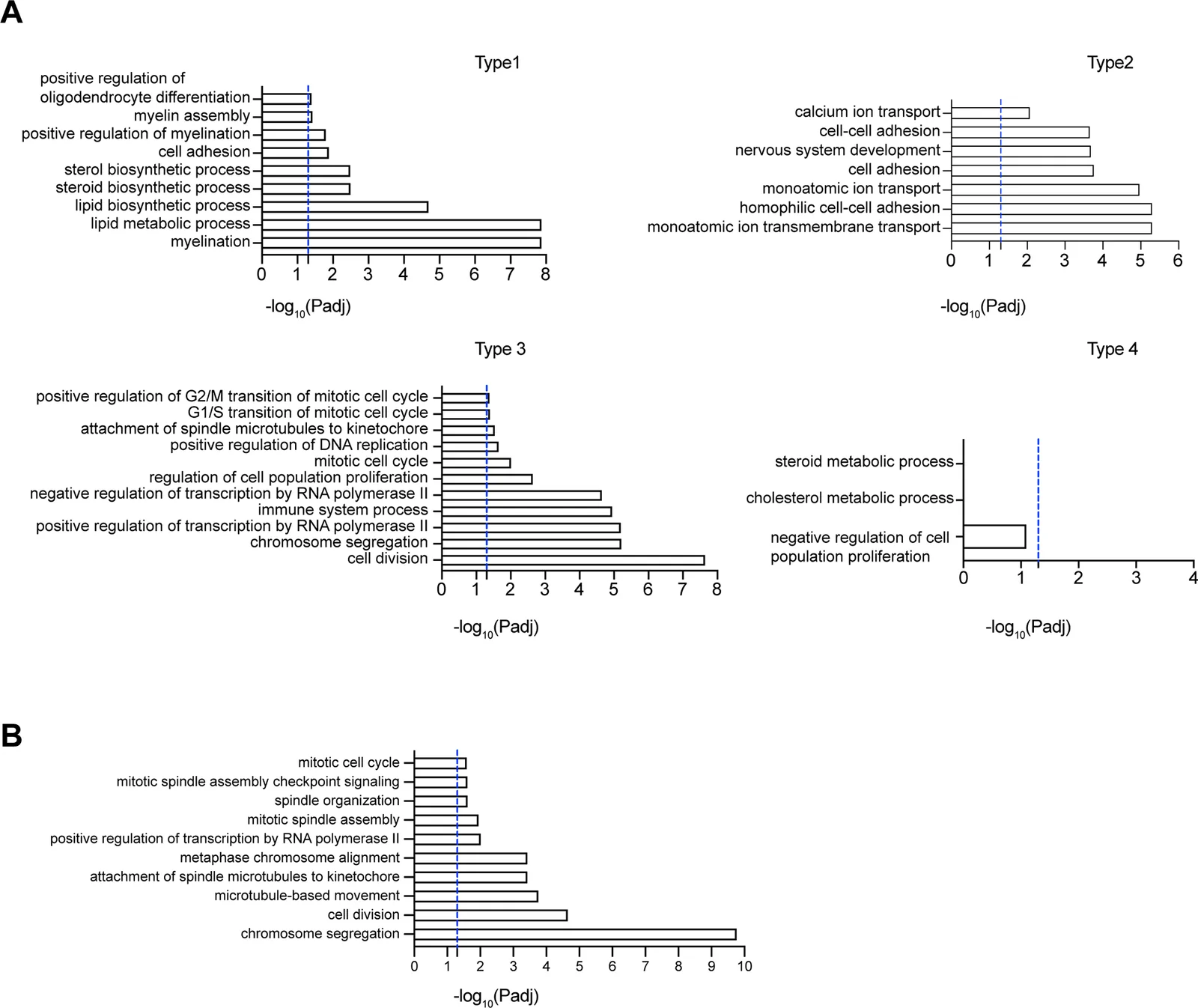
Gene ontology (GO) analysis. (**A**) Top over-represented GO terms for each switch type shown in Figure 1C. (**B**) Top over-represented GO terms for the 135 overlapping genes shown in Figure 2C, defined as genes that belong to type 3 switches and are also affected by ageing. P_adj_: Benjamini–Hochberg adjusted p-value. The dotted blue line indicates the significance threshold of P_adj_ = 0.05, corresponding to −log_10_(P_adj_)=1.3. Please note that only 8 type 4 genes overlapped with differentially expressed genes in ageing. Due to this small number, we could not identify any significant GO-term enrichment, and therefore this was not plotted. Figure 1—figure supplement 3—source data 1.Numerical data underlying Figure 1—figure supplement 3B.

To identify critical genes during oligodendrocyte lineage progression, we developed gSWITCH application (see Methods and Figure 1—figure supplement 2). Existing computational tools, such as Monocle (Trapnell et al., 2014), tradeSeq (Van den Berge et al., 2020), and maSigPro (Nueda et al., 2014), are highly valuable for identifying genes with dynamic expression changes across pseudotime or time-course data. gSWITCH addresses a different question. It does not aim to infer trajectories. It works with a user-defined ordered series of biological states or time points and asks a more specific question – does this gene show a statistically supported ‘switch-like’ change in expression as cells move through these states, and if so, what shape does that change take? It combines GLM-based statistical testing with criteria that capture where a gene reaches its highest or lowest expression and whether its expression changes steadily in one direction across the ordered series. To our knowledge, no existing tool combines significance testing with this type of explicit, shape-based classification into discrete, interpretable switch categories. gSWITCH sorts genes into four biologically meaningful patterns, rather than producing only a ranked list of significant genes based on pairwise comparisons between multiple conditions or states. gSWITCH also flags which of these switch genes are transcription factors, making it easier to prioritise candidates for follow-up experiments.

This biologist-friendly tool is freely available as a web application requiring no programming, works with experimental designs containing three or more stages or time points with at least two replicates per stage (no upper limit on either), and can be applied to bulk RNA-seq or to single-cell RNA-seq data aggregated as pseudobulk.

We identified four distinct dynamic patterns of gene expression, termed type 1 to type 4 switches (Figure 1C, Supplementary file 1, Figure 1—figure supplement 3A), during OL-lineage progression. These patterns are defined by the relative expression levels of specific gene sets across different cellular states. Disruptions to these expression levels can impair the coordinated dynamics and hinder lineage progression.

Among the four expression patterns, types 3 and 4 are most characteristic of OPCs, as they show higher relative expression in OPCs than in the next lineage stage, PreOL. For Type 4, expression levels rise again from PreOL to OL, but note that they do not return to the levels observed in OPCs. Indeed, gSWITCH is designed to detect a statistically significant difference between the first and last cell states (here, OPC vs OL), based on a user-defined fold-change threshold (1.5 in our case) (Figure 1—figure supplement 2).

### Altered expression of switches in aged OPCs

**Figure 2.**
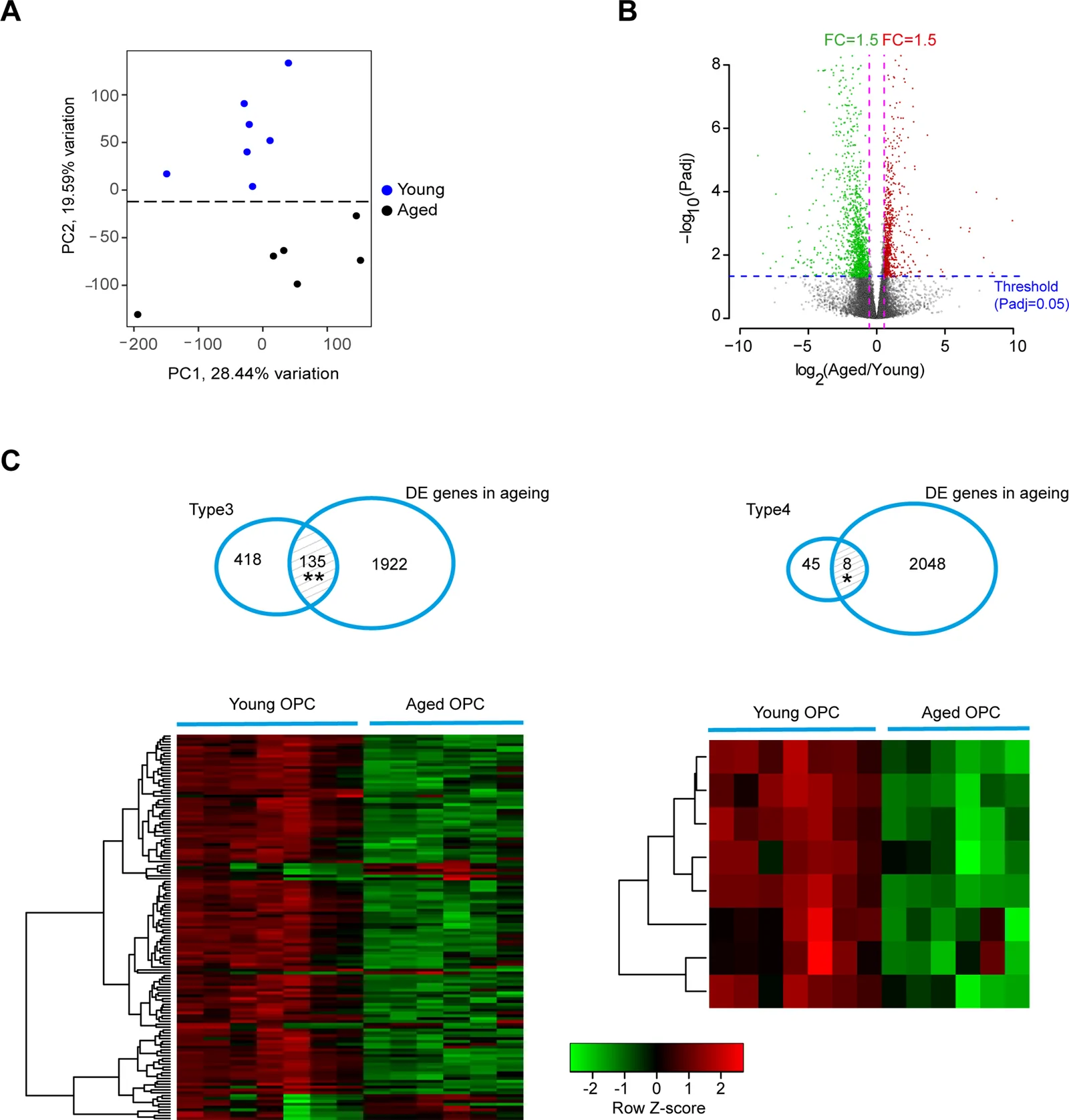
Down-regulation of switch genes due to ageing. (**A**) Principal component analysis (PCA) shows that aged and young oligodendrocyte progenitor cells (OPCs) are distinguishable based on global gene expression (meta-analysis of RNA-seq datasets, GEO accession number: GSE303317). (**B**) Volcano plot showing differentially expressed genes in aged OPCs compared to young OPCs. n=6 (aged rats), 7 (young rats), FC: fold change; Padj: adjusted p-value (Benjamini–Hochberg FDR after Wald test). (**C**) Venn diagram (top) showing the subset of type 3 and type 4 switch genes affected by ageing. Their expression patterns are shown in the heat map (bottom), where we observed down-regulation with ageing. *p<0.05, **p<0.001, Chi-square test (two-sided) with Yates’ correction. DE: Differentially expressed. DE gene list is accessible through GEO accession: GSE303317. See also Supplementary file 1, Figure 1—figure supplement 3B.

We next examined the regulation of type 3 and type 4 switches in aged OPCs. Our transcriptome meta-analysis showed clear differences between aged and young OPCs, identifying a set of genes that are consistently upregulated or downregulated in aged OPCs (Figure 2A, Figure 2B). A significant proportion of type 3 and type 4 switches were among these differentially expressed genes (135 out of 418 type 3 genes; 8 out of 45 type 4 genes) (Figure 2C, Figure 1—figure supplement 3B). Notably, these switches were significantly downregulated in aged OPCs (Figure 2C), suggesting that altered regulation of these critical genes may impair the differentiation of aged OPCs into OLs.

### *Bcl11a* is identified as a potential master switch

The orchestrated action of transcription factors (TFs) gradually drives the developmental program (Spitz and Furlong, 2012). Using gSWITCH, we identified switches that are TFs and found 67 TFs among type 3 and type 4 switches (Supplementary file 2).

**Figure 3.**
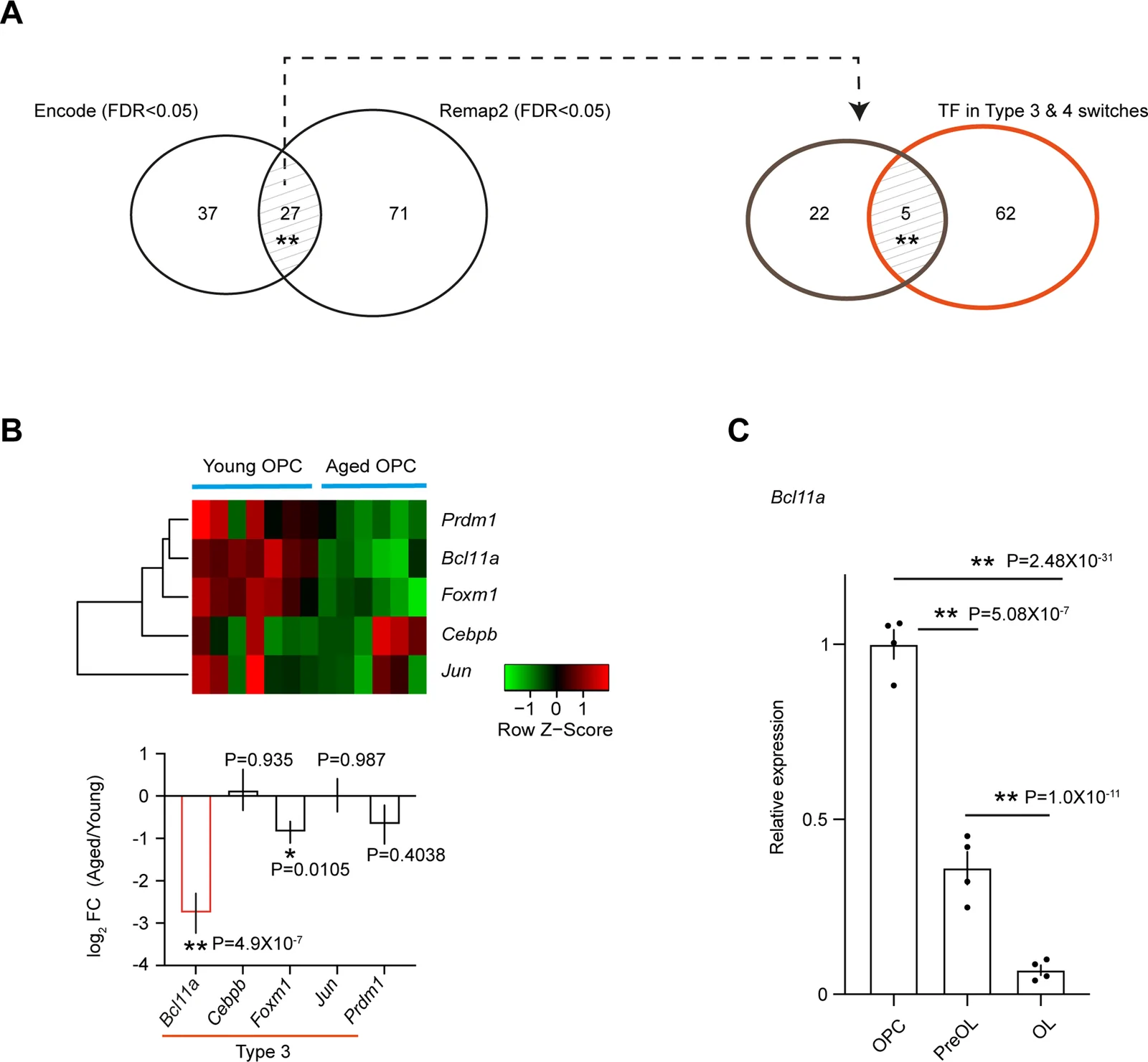
Transcription factor BCL11A-mediated network potentially affected during ageing. (**A**) Transcription factor binding site enrichment analysis using type 3 and type 4 switch genes as input identified 27 transcription factors (TFs) detected in both ENCODE and ReMap2, which are ChIP-seq–based experimental databases. Among these 27 TFs, we found 5 TFs that are themselves present as type 3 or type 4 switches (TF switches extracted from gSWITCH). **p<0.001, hypergeometric test. (**B**) Heat map (top) showing expression (RNA-seq, GEO accession number: GSE303317) of these 5 TFs. Their differential expression in aged oligodendrocyte progenitor cells (OPCs) relative to young OPCs plotted as log_2_FC (bottom). FC: Fold change, n=6 (aged rats), 7 (young rats), mean ± SEM, * adjusted p-value <0.05, ** adjusted p-value <1×10⁻⁶, Benjamini–Hochberg false discovery rate (FDR) after Wald test. Note: All five TFs are classified as type 3 switches, and *Bcl11a* shows the strongest repression in aged OPCs, with the highest statistical significance. (**C**) *Bcl11a*, as a type 3 TF switch, shows significantly repressed expression as oligodendrocyte (OL) lineage progresses. DESeq2-normalised values relative to OPCs plotted. n=4 different rats, mean ± SEM, ** adjusted p-value <1×10⁻⁶, Benjamini–Hochberg FDR after Wald test. GEO accession number of RNA-seq data: GSE301578. See also Supplementary file 2, Supplementary file 3, Figure 3—figure supplement 1. Figure 3—source data 1.Numerical data underlying Figure 3B and C.

Because a single transcription factor (TF) can regulate the expression of many genes, we aimed to identify TFs that may regulate type 3 and type 4 switches. TF binding site enrichment analysis using two chromatin immunoprecipitation (ChIP)-sequencing experimental evidence-based databases, ENCODE and ReMap2, predicted 27 TFs common to both datasets (Figure 3A, Supplementary file 3). We then examined whether any of these 27 TFs were present in type 3 and type 4 switches and identified five [Zinc finger protein B-cell CLL/Lymphoma 11 A (*Bcl11a*) also known as *Ctip1* (COUP-TF–interacting protein 1), CCAAT/enhancer-binding protein beta (*Cebpb*), Forkhead box M1 (*Foxm1*), *Jun* Proto-Oncogene, AP-1 Transcription Factor Subunit (*Jun*), and PR Domain Zinc Finger Protein 1 (*Prdm1*)], all of which were defined as type 3 switches (Figure 3A, Figure 3B, Supplementary file 3). Among these, only *Bcl11a* and *Foxm1* were significantly downregulated in aged OPCs (Figure 3B). We focused further on *Bcl11a*, as its magnitude of change being greater than that of *Foxm1* (Figure 3B).

**Figure 3—figure supplement 1.**
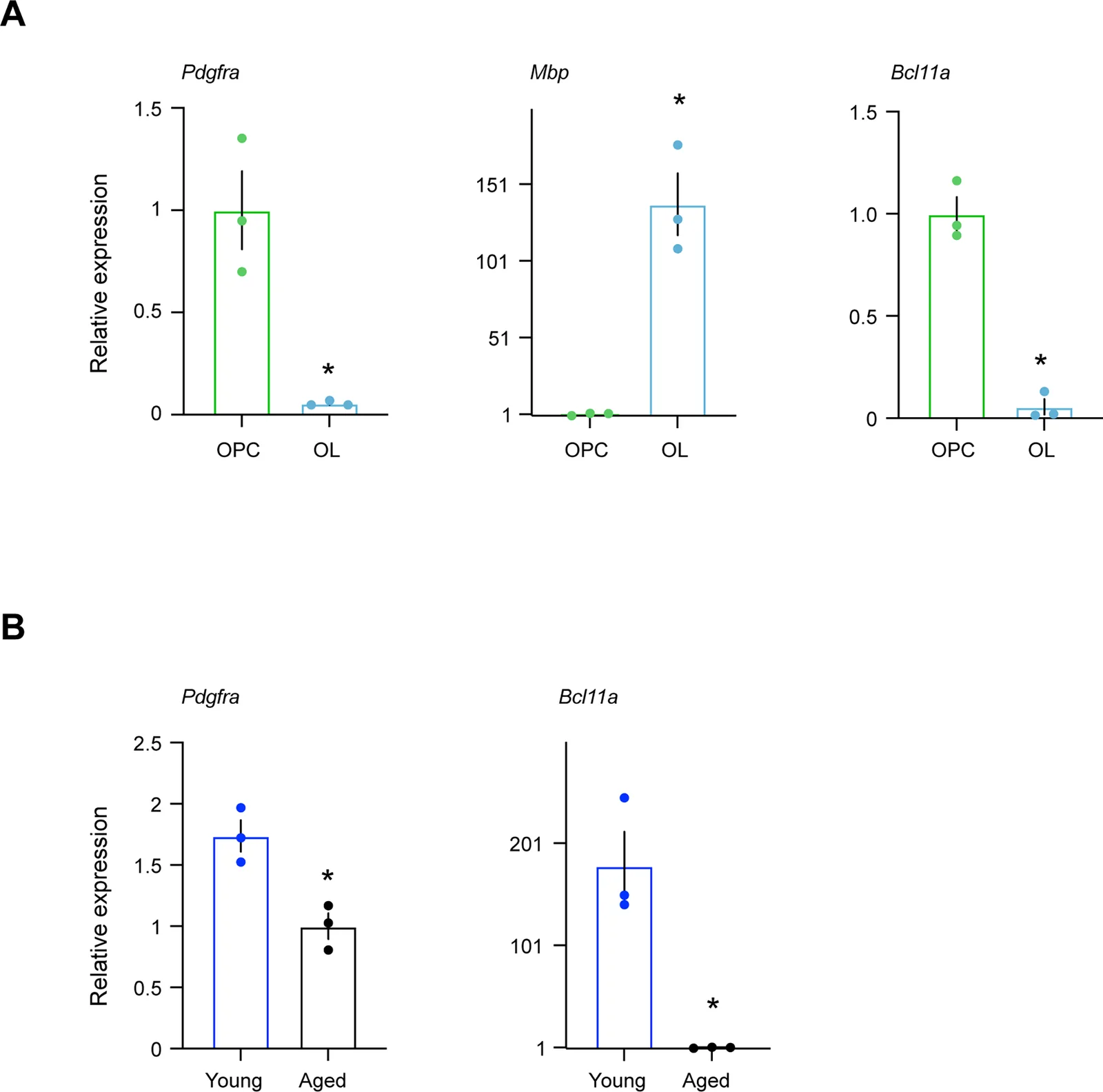
*Bcl11a* expression in oligodendrocyte progenitor cells (OPCs), oligodendrocytes (OLs), and aged OPCs, as determined by RT-qPCR (reverse transcription followed by qPCR). (**A**) Expression levels of *Bcl11a*, *Pdgfra* as an OPC marker, and *Mbp* as an OL marker were measured in OPCs and OLs and plotted relative to OPCs. (**B**) Expression levels of *Bcl11a* and *Pdgfra* were measured in young and aged OPCs and plotted relative to aged OPCs. (**A, B**) n=3 rats, mean ± SEM, * Holm-adjusted p<0.05, Welch’s t-test (unpaired, two-tailed). Figure 3—figure supplement 1—source data 1.Numerical data underlying Figure 3—figure supplement 1A and B.

The expression of *Bcl11a* followed a typical type 3 pattern: it was highest in OPCs and gradually decreased during differentiation, reaching significantly lower levels in OLs (Figure 3C, Figure 3—figure supplement 1A). We, therefore, experimentally tested whether *Bcl11a* plays a functional role in OPC-to-OL differentiation, and whether restoring its expression can rescue the impaired differentiation capacity of aged OPCs.

### Inhibition of *Bcl11a* in OPCs markedly reduced their differentiation

**Figure 4.**
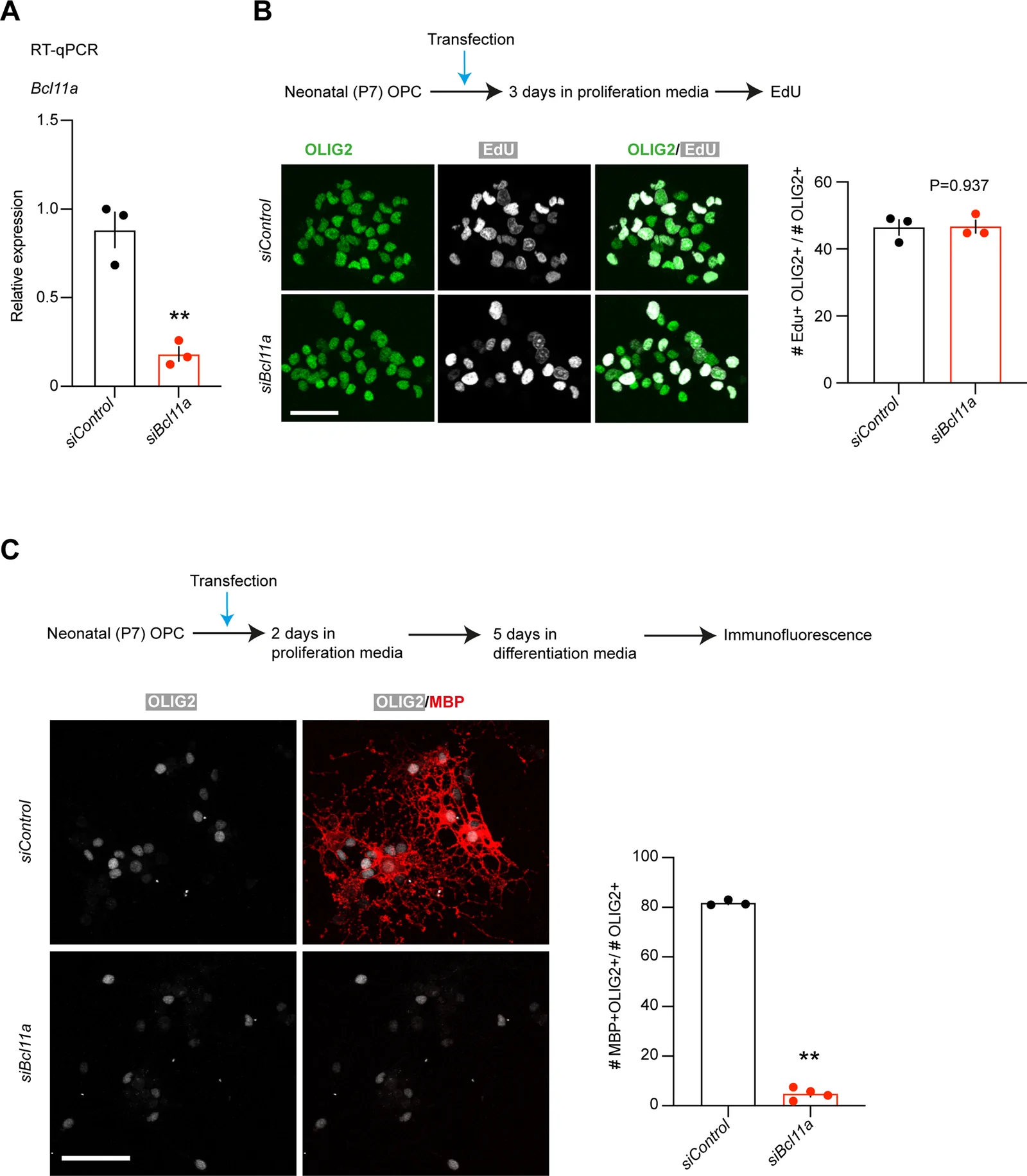
Effect of *Bcl11a* inhibition on oligodendrocyte progenitor cells (OPCs) proliferation and differentiation. (**A**) Reverse transcription (RT)-qPCR analysis of *Bcl11a* expression in OPCs transfected with siRNA targeting *Bcl11a. siBcl11a*: siGENOME Rat *Bcl11a; siControl*: siGENOME non-targeting control siRNA (Dharmacon). (**B**) **Effect on proliferation**. Left: Immunofluorescence analysis using antibodies to OLIG2 and EdU detection (Click-iT protocol), followed by EdU proliferation assay. Right: Quantification of EdU^+^OLIG2^+^ cells plotted as a percentage of OLIG2^+^ cells. P: p-value, Student’s t-test (unpaired and two-tailed) (**C**) **Effect on differentiation**. Left: Immunofluorescence analysis using antibody to myelin basic protein (MBP), OLIG2 upon inhibition of *Bcl11a* in OPCs. Right: quantification of MBP+ cells were plotted as a percentage of OLIG2+ cells. (**A–C**) n=3 independent experiments (each time 4 P7 brains were pooled for OPC isolation). mean ± SEM. **p<0.01, Student’s t-test (unpaired, two-tailed); The symbol ‘#’ indicates cell count. (**B, C**) Scale bar: 22 µm. Figure 4—source data 1.Numerical data underlying Figure 4A–C.

Since *Bcl11a* expression decreases as OPCs differentiate into OLs (Figure 3C, Figure 3—figure supplement 1A), we addressed the hypothesis that, if *Bcl11a* acts as a master switch for lineage progression, its early expression in OPCs would be functionally required for differentiation to proceed. To test this, we used small interfering RNA (siRNA)-mediated knockdown. Transfection with siRNA targeting *Bcl11a* (*siBcl11a*) reduced its expression by approximately 79% in proliferating OPCs (Figure 4A). Using the 5-ethynyl-2'-deoxyuridine (EdU) incorporation assay, we found no significant effect of *Bcl11a* knockdown on OPCs proliferation (Figure 4B). However, *Bcl11a* inhibition led to a substantial (~77%) reduction in differentiation, as measured by immunostaining for myelin basic protein (MBP), a marker of OL differentiation (Figure 4C). These findings suggest that as a type 3 switch *Bcl11a* function is essential for priming OPCs for efficient differentiation.

### Transient overexpression of *Bcl11a* in aged OPCs improves differentiation

**Figure 5.**
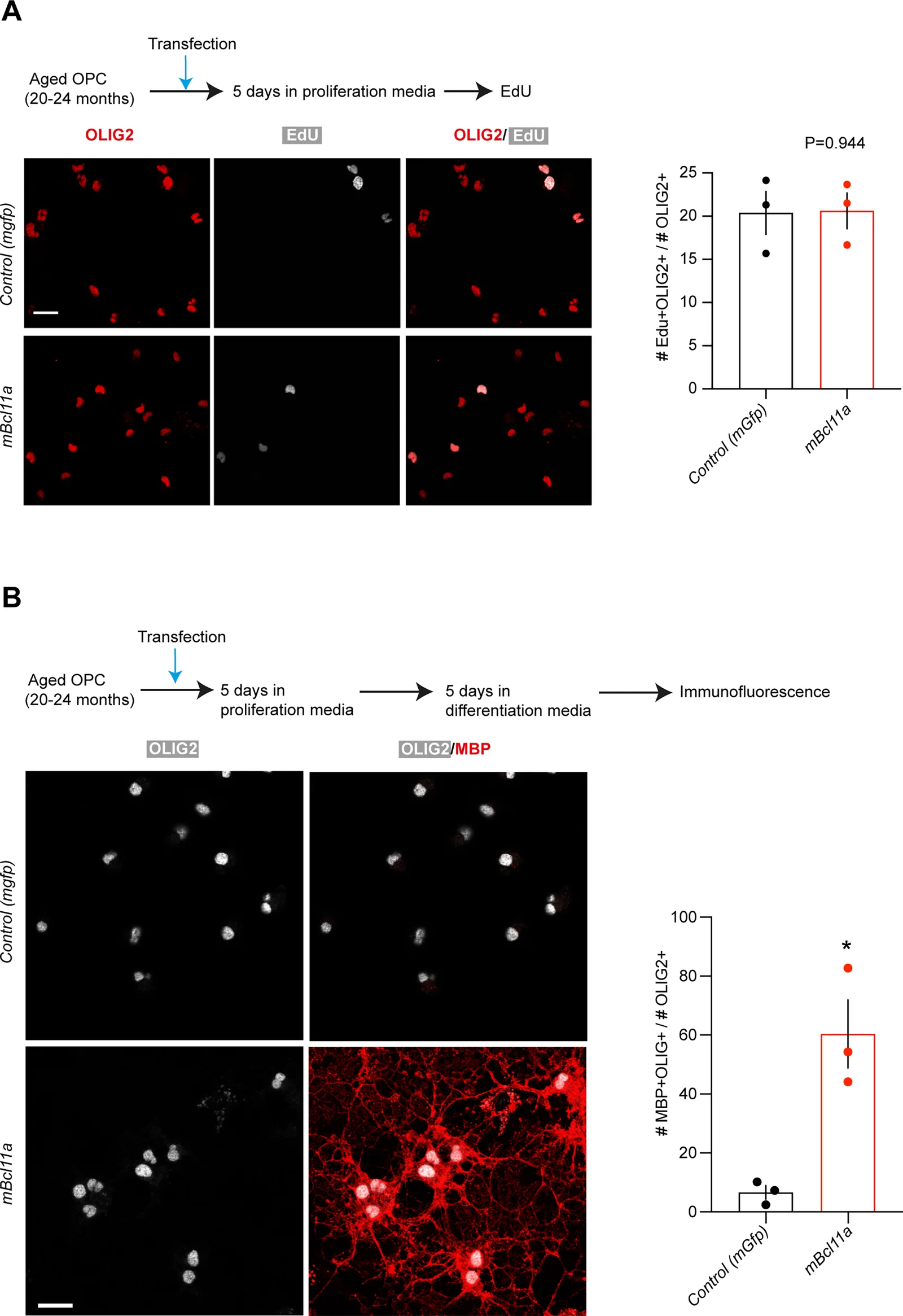
Effect of *Bcl11a* transient over-expression on proliferation and differentiation of aged oligodendrocyte progenitor cells (OPCs). (**A**) **Effect on proliferation**. Left: Immunofluorescence analysis using antibodies to OLIG2 and EdU detection (Click-iT protocol), followed by EdU proliferation assay. Right: Quantification of EdU^+^OLIG2^+^ cells plotted as a percentage of OLIG2^+^ cells. P: p-value, Student’s t-test (unpaired, two-tailed). (**B**) **Effect on differentiation**. Left: Immunofluorescence analysis using antibodies to myelin basic protein (MBP) and OLIG2 after transfection of *Bcl11a* mRNA (m*Bcl11a*) or *Gfp* mRNA (m*Gfp*; control) in aged OPCs. Right: Quantification of MBP^+^ cells plotted as a percentage of OLIG2^+^ cells. *p<0.05, Student’s t-test (unpaired, two-tailed). (**A, B**) n=3 independent experiments (each using OPCs isolated from pooled brains of two aged rats). mean ± SEM. The symbol ‘#’ indicates cell count. Scale bar: 22 µm. See also Figure 5—figure supplement 1A and B. Figure 5—source data 1.Numerical data underlying Figure 5A and B.

As noted, our RNA-seq analysis revealed reduced *Bcl11a* expression in aged OPCs (Figure 3B, Figure 3—figure supplement 1B). To investigate whether restoring *Bcl11a* levels could overcome their impaired differentiation capacity, we expressed *Bcl11a* in aged OPCs (isolated from 20 to 24 month-old rats). We first performed in vitro transcription to generate *Bcl11a* mRNA (*mBcl11a*) and confirmed the fidelity of its protein expression by western blot analysis (Figure 5—figure supplement 1A). m*Bcl11a* was subsequently transfected into aged OPCs; *Gfp* mRNA (*mGfp*) served as a control. Transfection efficiency was approximately 77% (Figure 5—figure supplement 1B). Similar to *Bcl11a* knockdown, *mBcl11a* expression had no significant effect on aged OPC proliferation (Figure 5A). However, culturing these OPCs in differentiating media led to an approximately 54% increase in OL numbers (Figure 5B), indicating that *Bcl11a* expression in OPCs is critical for their progression toward the oligodendrocyte lineage.

**Figure 5—figure supplement 1.**
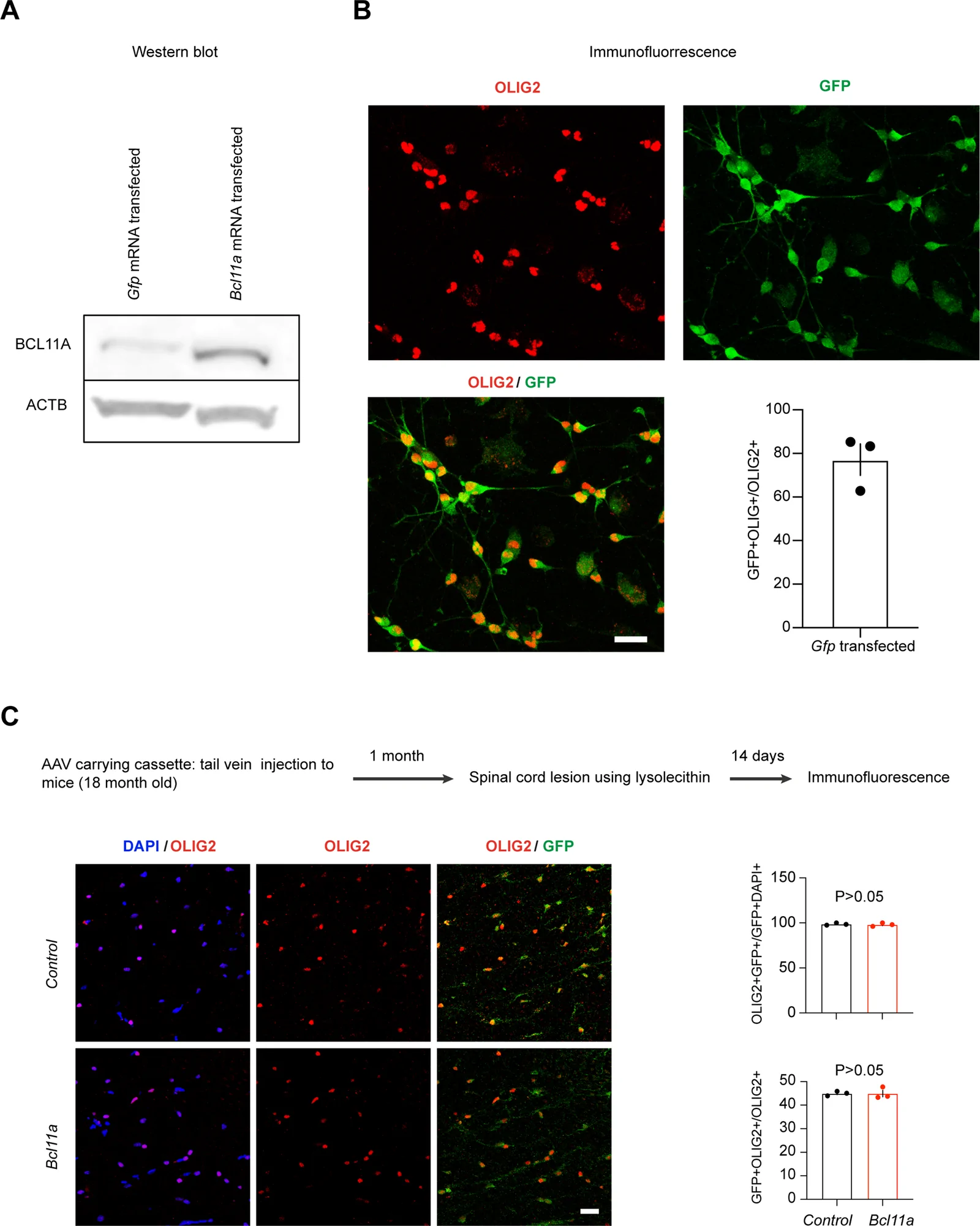
Fidelity of protein expression from in vitro–transcribed *Bcl11a* mRNA; mRNA transfection efficiency in aged oligodendrocyte progenitor cells (OPCs); and fidelity of adeno-associated virus (AAV) construct expression in the oligodendrocyte (OL) lineage and infection efficiency in aged mouse brain. (**A**) Western blot analysis using antibodies to BCL11A and β-actin (ACTB), with ACTB serving as a loading control, in MCF7 cells transfected with *Bcl11a* mRNA or *Gfp* mRNA (control). The molecular weights of BCL11A and ACTB are approximmately 91 KDa and 42 KDa, respectively. (**B**) Immunofluorescence analysis using antibodies to OLIG2 and GFP in aged OPCs transfected with *Gfp* mRNA. Quantification of GFP+ OLIG2+ cells were plotted as percentage of OLIG2+ cells. n=3 independent experiments (each using OPCs isolated from pooled brains of two aged rats). mean ± SEM. Scale bar: 22 µm. (**C**) AAV-carrying constructs are shown in Figure 6A. Left: Immunofluorescence analysis of OLIG2 and GFP immunostaining. Right top: Quantification of OLIG2+ GFP+ cells was plotted as a percentage of GFP+ DAPI+ cells. Right bottom: quantification of OLIG2+ GFP + cells were plotted as a percentage of OLIG2+ cells. n=3 mice, mean + SEM. P: p-value, Student’s t-test (unpaired, two-tailed). Scale bar: 20 µm. Figure 5—figure supplement 1—source data 1.Numerical data underlying Figure 5—figure supplement 1B and C. Figure 5—figure supplement 1—source data 2.Original western blot images corresponding to Figure 5—figure supplement 1A. Figure 5—figure supplement 1—source data 3.Original western blot images corresponding to Figure 5—figure supplement 1A, with relevant bands labelled.

**Figure 6.**
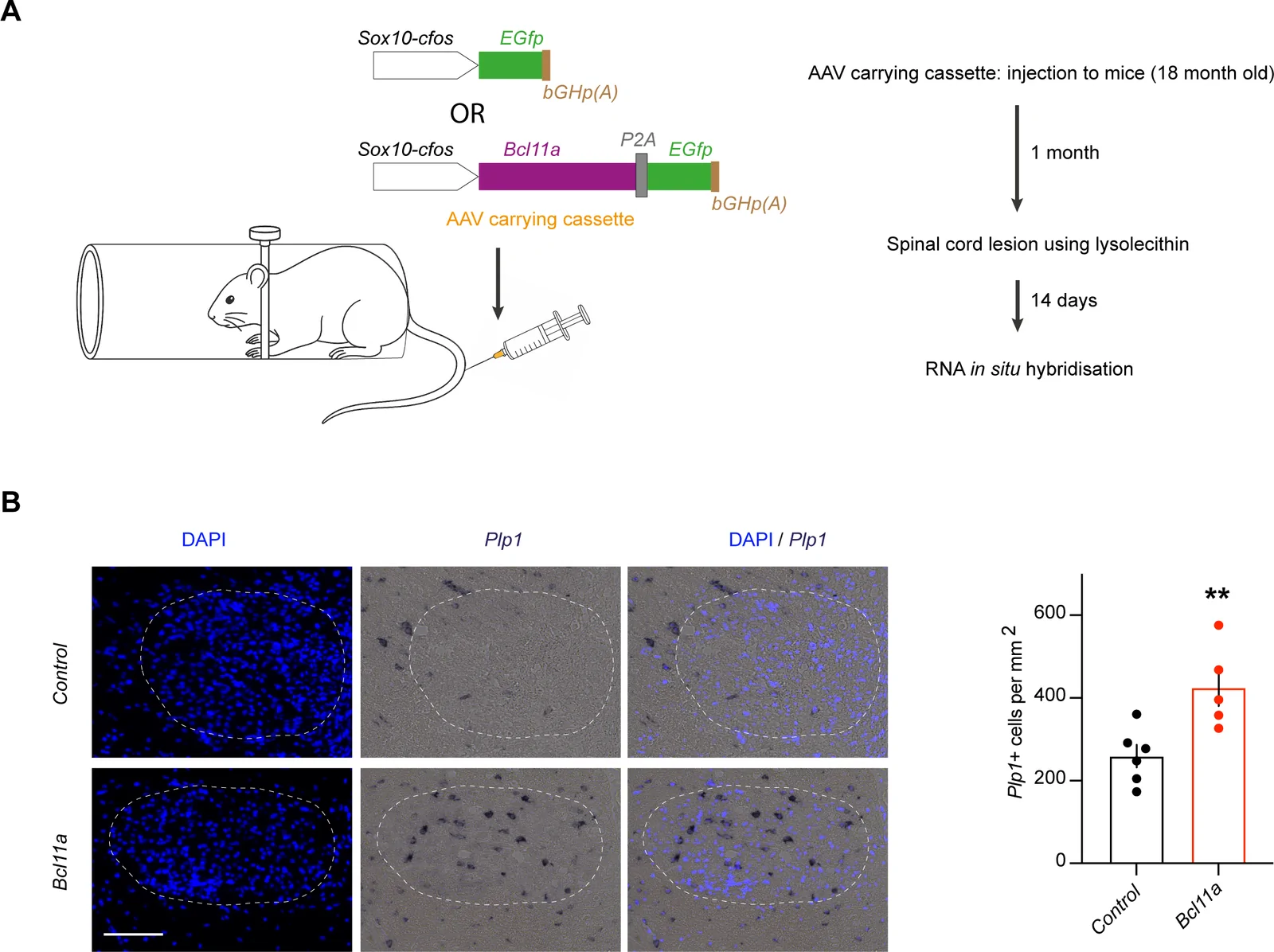
Oligodendrocyte (OL) lineage specific *Bcl11a* expression in aged mice. (**A**) Left: *Sox10*-driven construct carrying either *Gfp* alone (control) or *Bcl11a* linked to *Gfp* via a self-cleaving peptide element (*P2A*). This construct ensures *Sox10*-driven expression of both *Bcl11a* and *Gfp*. P2A: Porcine teschovirus-1 2 A peptide, *bGH p(A*): bovine growth hormone poly(A) signal. The adeno-associated virus (AAV) containing this construct was delivered to 18-month-old mice via tail-vein injection. Right: schematic showing the experimental timeline. (**B**) RNA in situ analysis of *Plp1*. In the lesioned area, the number of *Plp1*^+^ cells per mm² was counted and plotted. n=5–6 mice, mean ± SEM, **p<0.01, Student’s t-test (unpaired, two-tailed). Scale bar: 100 µm. The white dotted line demarcates the lesion area. See also Figure 5—figure supplement 1C, Figure 6—figure supplement 1. Figure 6—source data 1.Numerical data underlying Figure 6B.

We next tested the effect of *Bcl11a* expression in OPCs in aged mice (18-month-old) using a model of focal demyelination in spinal cord white matter. We injected an adeno-associated virus (AAV) carrying a *Sox10*-driven *Bcl11a-P2A-gfp* construct into the tail vein of 18-month-old mice (Figure 6A). This design ensures *Sox10*-driven expression of both *Bcl11a* and *Gfp* via a self-cleaving P2A peptide. As a control, we injected AAV carrying a *Sox10*-driven *Gfp* construct. One month after AAV injection, we induced spinal cord lesions using lysolecithin. Fourteen days later, the spinal cords were harvested. We found that nearly all GFP immune-positive cells were also OLIG2-positive (98.6% in controls, total GFP+ 4’,6-diamidino-2-phenylindole [DAPI]+ cells counted: 193; 98% in *Bcl11a*, total GFP+ DAPI+ cells counted: 163), indicating strong oligodendrocyte lineage fidelity (Figure 5—figure supplement 1C). Our protocol infected approximately 45% of GFP+ OLIG2+ cells (190 GFP+ OLIG2+ out of 422 OLIG2 + cells counted in control; 160 GFP+ OLIG2+ out of 357 OLIG2+ cells counted in *Bcl11a*) (Figure 5—figure supplement 1C). We performed RNA in situ hybridisation using a probe specific for proteolipid protein 1 (*Plp1*), a marker of late oligodendrocyte differentiation. We observed no difference in total OLIG2+ cells in the lesion area but a significantly increased number of OLIG2+ cells that were *Plp1*+ in *Bcl11a* samples (Figure 6—figure supplement 1; Figure 6B), suggesting *Bcl11a* restores the differentiation capacity of aged OPCs recruited to the injury area.

**Figure 6—figure supplement 1.**
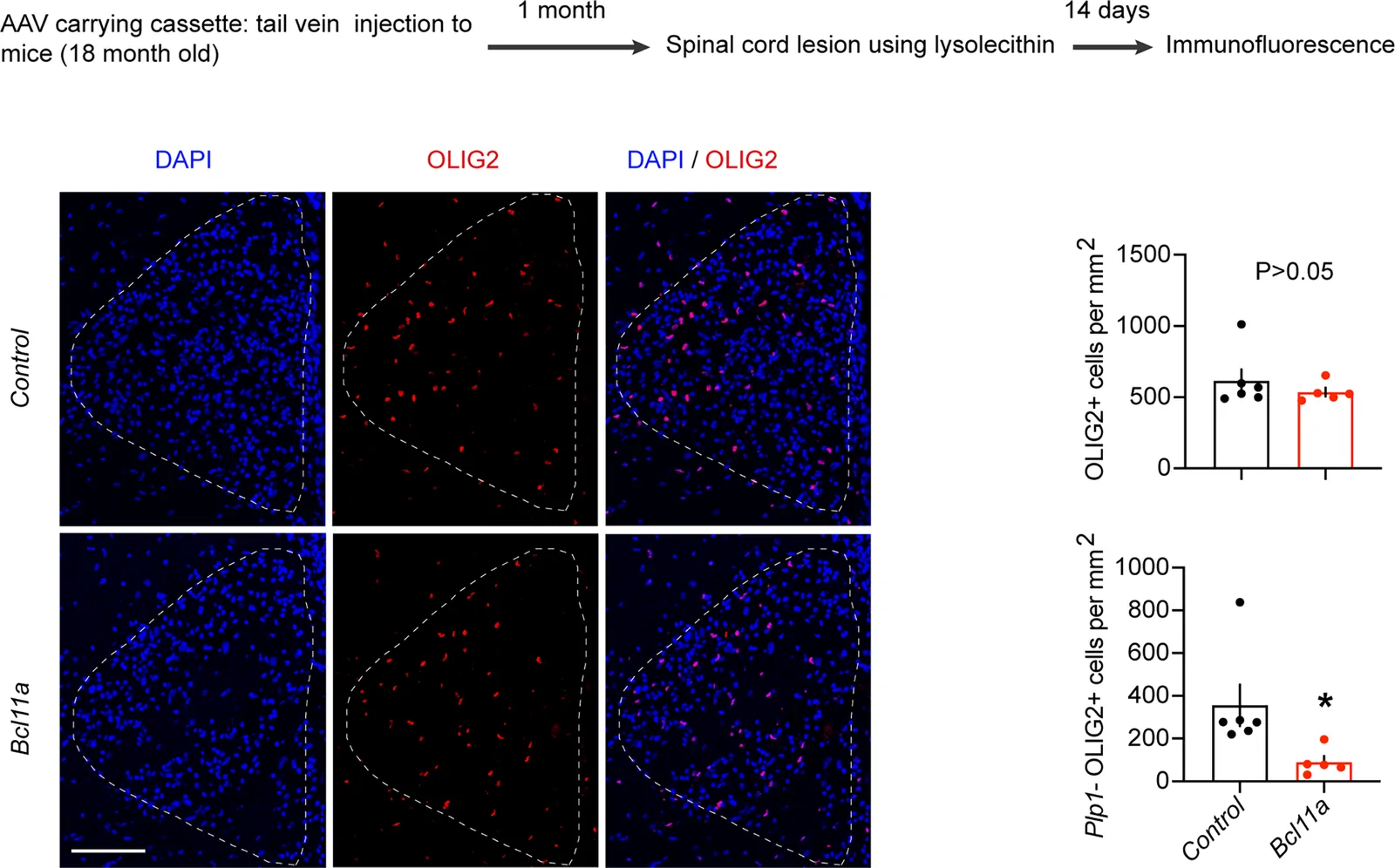
Immunofluorescence analysis using antibody to OLIG2 after oligodendrocyte (OL) lineage specific *Bcl11a* expression in aged mice. The adeno-associated virus (AAV) containing the *Sox10*-driven construct carrying either *Gfp* alone (control) or *Bcl11a* linked to *Gfp* via a self-cleaving peptide element (P2A) was delivered by tail-vein injection (see Figure 6A). In the lesioned area, the number of OLIG2+ cells per mm^2^ was counted and plotted. In the same region, the number of *Plp1*⁻ OLIG2^+^ cells per mm² was also calculated and plotted (see Figure 6B for *Plp1*^+^ cells per mm²). n=5–6 mice, mean ± SEM, P: p-value, *p<0.05, Student’s t-test (unpaired, two-tailed). Scale bar: 100 µm. The white dotted line demarcates the lesion area. Figure 6—figure supplement 1—source data 1.Numerical data underlying Figure 6—figure supplement 1.

### *Bcl11a* has undergone purifying selection during vertebrate evolution

Given that *Bcl11a* functions as an important regulatory transcription factor switch during oligodendrocyte lineage progression and that its expression level is associated with functional vulnerability in ageing, we used computational molecular evolution approaches to investigate whether selective constraints during evolution have acted on its codon sequence. We included twenty-three vertebrate species, including humans (Supplementary file 4). We aimed to determine whether such constraints have preserved the amino acid sequence by eliminating deleterious mutations through purifying selection. *Bcl11a* is absent in invertebrate genomes (UCSC Genome Browser search), but orthologs are present across all examined vertebrate species. The ratio of nonsynonymous (dN) to synonymous (dS) substitution rates – known as the dN/dS ratio – is a widely used metric in molecular evolution to evaluate the selective pressures on protein-coding genes. (Yang, 2007; Anisimova and Kosiol, 2009). As shown in Table 1, our analysis using multiple evolutionary models consistently yielded dN/dS values below 1, providing strong evidence of pervasive purifying selection acting on *Bcl11a* across the vertebrate lineage.

**Table 1.** Test for evolutionary selection on Bcl11a.

Model	Description	Parameter estimates and test statistics	Interpretation
M0	M0 (one ratio): assumes that all sites have the same dN/dS.	dN/dS=0.064 (SE: 0.0023) logL=–21196.13	M0 suggests pervasive purifying selection
M3 compared with M0	M3 (discrete): permits 3 different types of sites with different dN/dS values. Comparison of models M0 and M3 is a test of whether there is variation of selective pressures over sites, including the possibility of sites with dN/dS >1.	Compared with M0: dlogL=–20788.43 - (–21196.13)=407.7 χ^2^ test (4 df) indicates significant preference for M3 (p<0.00001)	LRT indicates M3 significantly preferred to M0, i.e., strong evidence of selective pressures varying over sites. All sites under purifying selection
M2a compared with M1a	M1a (neutral): assumes 2 types of sites, one with dN/dS <1 and one with dN/dS=1. M2a (selection): in addition to the categories in M1a, includes a third class of sites with dN/dS >1. Comparison of these models is a test of whether there are any sites with dN/dS >1.	dlogL=–20983.51- (–20983.62)=0.11 χ^2^ test (2 df) indicates significant preference for M1a (p=0.90)	Absence of positive selection.

Note: dN: number of non-synonymous changes per non-synonymous site, dS: number of synonymous changes per synonymous site, SE: Standard error, logL: log-likelihood value, dlogL: log-likelihood difference, LRT: likelihood ratio test (2dlogL compared with appropriate χ^2^ distribution).

## Discussion

Dynamically regulated genes during transitions from one cellular stage to another offer important information about the regulatory mechanisms governing differentiation (Spitz and Furlong, 2012; Pope and Medzhitov, 2018). Our gSWITCH web application is a powerful tool for identifying specific dynamic patterns based on progressive changes in gene expression during cellular state transitions or any variable-dependent changes. This can be particularly valuable for discovering novel mechanisms in defined biological contexts. Switch genes signify the neurogenic commitment of cortical progenitors and have enabled the identification of new regulators of brain corticogenesis (Aprea et al., 2013). However, no biologist-friendly tool has been available to systematically detect such genes. gSWITCH is a biologically inspired method, and here, we experimentally validate its prediction.

Using gSWITCH and following a data-driven approach, we identified *Bcl11a* as a critical switch gene in the transition from OPCs to pre-OLs. To test this prediction, we performed loss-of-function (LOF) experiments using siRNA and found that inhibition of *Bcl11a* impaired OPC differentiation into oligodendrocytes. Because *Bcl11a* expression is markedly down-regulated in aged OPCs, we next carried out gain-of-function (GOF) experiments both in vitro and in vivo. Restoring *Bcl11a* expression in aged OPCs significantly enhanced their differentiation. Together, the LOF and GOF experiments unambiguously validate the key role of *Bcl11a* in oligodendrocyte lineage progression and support the gSWITCH prediction.

The extent to which the reduced differentiation capacity of aged OPCs is intrinsically encoded within the cells themselves or induced by prolonged exposure to an aged tissue environment is an interesting question. Based on our previous work, we favour the view that loss of OPC function is primarily determined extrinsically since various manipulations of the aged environment, such as heterochronic parabiosis (Ruckh et al., 2012), fasting, and calorie restriction mimetics (Neumann et al., 2019), and niche biomechanics (Segel et al., 2019), can all alter the cell-intrinsic state, reverting aged cells to a ‘youthful state’. Significantly, when aged OPCs are transplanted into the neonatal CNS, they proliferate and differentiate as if they were neonatal OPCs (Segel et al., 2019). The reversion of aged OPCs to a functional state by changes in their external environment necessarily operates through changes in cell-intrinsic function, suggesting that the same intrinsic mechanisms could be targeted directly to restore declining OPC function—for example through epigenetic regulation of differentiation inhibitors (Shen et al., 2008) or overexpression of transcriptional regulators, such as *c-Myc* (Neumann et al., 2021; Dimas et al., 2025).

Ageing is undeniably complex, with multiple cellular, metabolic, and extracellular changes, and inflammation occurring in parallel. Discovering new regulators remains essential for understanding how these layers collectively shape age-related decline. Interventions used so far have shown that no single mechanism can fully explain the functional deterioration of ageing oligodendrocyte progenitors. Instead, each factor highlights a distinct point at which the ageing process exerts pressure, gradually revealing the distributed architecture through which ageing unfolds. Within this landscape, our identification of *Bcl11a –* emerging from an analysis of oligodendrocyte lineage progression – defines another biologically meaningful regulatory axis involved in these age-associated changes, expanding the mechanistic resolution with which ageing can be understood. Only by charting these individual components can we explain how diverse age-related diseases emerge and begin to design personalised strategies capable of addressing the true complexity of ageing in a mechanistically grounded way.

*Bcl11a* encodes a zinc-finger transcription factor primarily known as a transcriptional repressor, though it can also function as an activator (Horton et al., 2025; Bauer and Orkin, 2015; Lee et al., 2018). Our computational molecular-evolution analyses indicate that *Bcl11a* is a vertebrate-specific transcription factor that has undergone pervasive purifying selection. In this context, *Bcl11a* is perhaps evolutionarily linked to major cellular programs, and its dysfunction may contribute to disease. In haemoglobin regulation, *Bcl11a* orchestrates the fetal-to-adult haemoglobin switch by binding to and repressing the γ-globin genes, thereby enabling activation of adult β-globin – a critical transition that supports the oxygen-transport requirements of postnatal life (Liu et al., 2018). In oligodendrocytes, we find that *Bcl11a* is essential for lineage progression. *Bcl11a* expression in young OPCs is important for maintaining their functional resilience, ensuring they retain a strong capacity to differentiate efficiently.

Notably, *Bcl11a* deficiency or dysfunction has been implicated in autism spectrum disorder and intellectual disability based on human genetic studies (De Rubeis et al., 2014; Dias et al., 2016; Cai et al., 2017). In the hematopoietic system, *Bcl11a* deficiency in hematopoietic stem cells induces an ageing-like state (Luc et al., 2016). Consistent with this, we observed that aged OPCs, despite being impaired in their differentiation capacity, were able to regain functional resilience when *Bcl11a* expression was restored. In this sense, *Bcl11a* reinstates functional robustness in aged OPCs. Future studies will elucidate the detailed gene-regulatory mechanisms through which *Bcl11a* functions in young OPCs, clarify how it enhances functional competence in aged OPCs, and further define its potential as a therapeutic target.

## Materials and methods

**Key resources table:** 

Reagent type (species) or resource	Designation	Source or reference	Identifiers	Additional information
Antibody	anti-MBP (Rat monoclonal)	Serotec	Cat# MCA409S; RRID:AB_325004	IF (1:500 or 1:100)
Antibody	Anti-OLIG2 (Rabbit polyclonal)	Millipore	Cat# AB9610; RRID:AB_570666	IF (1:1000 or 1:100)
Antibody	Anti-BCL-11A (Rabbit monoclonal)	Abcam	Cat# ab191401; RRID:AB_2921236	Western blot (1:3000)
Antibody	Anti-beta Actin (mouse monoclonal)	Abcam	Cat# ab6276; RRID:AB_2223210	Western blot (1:3000)
Antibody	Anti-GFP (chicken polyclonal)	Abcam	Cat# ab13970; RRID:AB_300798	IF (1:1000)
Antibody	Cy3 AffiniPure Anti-Rat	Jackson Immuno Research	Cat# 712-165-150; RRID:AB_2340666	IF (1:1000)
Antibody	Alexa Fluor 647 Anti-Rabbit	Invitrogen	Cat# A31573; RRID:AB_2536183	IF (1:1000 or 1:500)
Antibody	Alexa Fluor 488 Anti-Chicken	Jackson Immuno Research	Cat# 703-546-155; RRID:AB_2340376	IF (1:1000)
Antibody	Alexa Fluor 488 anti-rabbit	Invitrogen	Cat# A11008; RRID:AB_143165	IF (1:1000)
Antibody	Alexa Fluor 594 anti-rabbit	Invitrogen	Cat# A11012; RRID:AB_2534079	IF (1:1000)
Antibody	HRP-conjugated donkey anti-rabbit IgG secondary	Amersham/GE Healthcare	Cat# NA934; RRID:AB_772206	Western blot (1:10000)
Antibody	HRP-conjugated sheep anti-mouse IgG secondary	Amersham/GE Healthcare	Cat# NA931; RRID:AB_772210	Western blot (1:10000)
Commercial assay or kit	Click-iT Plus EdU Alexa Fluor 647 Imaging Kit	Thermo Fisher Scientific	Cat# C10640	NA
Commercial assay or kit	pCR 2.1-TOPO TA using TOPO TA Cloning Kit	Thermo Fisher Scientific	Cat# K450001	NA
Cell line (*Homo sapiens*)	MCF7	ATCC	Cat# HTB-22	NA
Biological sample (*Rattus norvegicus*)	Oligodendrocyte progenitor cells[neonatal (P7), young (2–3 months), aged (20–24 months)]	Charles River Laboratories, Margate, UK	NA	Sprague–Dawley rats. See Materials and methods
Strain, strain background (*Mus musculus*)	C57BL/6 mice	Charles River Laboratories, Margate, UK	NA	See Materials and methods
Software, algorithm	gSWITCH	This paper	NA	https://github.com/Tanay-math/gSWITCH; https://altoslabs.shinyapps.io/gSWITCH/
Software, algorithm	Codeml	PMID:17483113	NA	http://abacus.gene.ucl.ac.uk/software/paml.html
Software, algorithm	MAFFT	PMID:23329690	RRID:SCR_011811	https://mafft.cbrc.jp/alignment/software/
Software, algorithm	PAL2NAL	PMID:16845082	NA	https://bioconda.github.io/recipes/pal2nal/README.html
Software, algorithm	DESeq2	PMID:25516281	RRID:SCR_015687	https://bioconductor.org/packages/release/bioc/html/DESeq2.html
Software, algorithm	limma	PMID:25605792	RRID:SCR_010943	https://bioconductor.org/packages/release/bioc/html/limma.html
Software, algorithm	ChEA3	PMID:31114921	RRID:SCR_023159	https://maayanlab.cloud/chea3/
Software, algorithm	Salmon	PMID:28263959	RRID:SCR_017036	https://github.com/COMBINE-lab/salmon
Software, algorithm	SortMeRNA	PMID:23071270	RRID:SCR_014402	https://github.com/sortmerna/sortmerna
Software, algorithm	Trimmomatic	PMID:24695404	RRID:SCR_011848	http://www.usadellab.org/cms/?page=trimmomatic
Software, algorithm	ImageJ version 2.1.0	PMID:22743772	RRID:SCR_003070	https://imagej.net
Sequence-based reagent	siGENOME Rat *Bcl11a* (305589)-SMARTpool (siRNA)	Dharmacon	Cat# M-085668-01-0010	See Materials and methods
Sequence-based reagent	siGENOME non-Targeting siRNA Pool #2 (siRNA)	Dharmacon	Cat# D-001206-14-05	See Materials and methods
Chemical compound, drug	Lipofectamine RNAiMAX	Thermo Fisher Scientific	Cat# 13778030	See Materials and methods
Chemical compound, drug	Lipofectamine MessengerMax	Thermo Fisher Scientific	Cat# LMRNA008	See Materials and methods
Recombinant DNA reagent	*Sox10-cfos-Bcl11a-P2A-GFP-bGH* (Plasmid)	This paper	NA	Construct for in vivo experiment. See Materials and methods
Recombinant DNA reagent	*Sox10-cfos-GFP-bGH* (Plasmid)	This paper	NA	Construct for in vivo experiment. See Materials and methods
Sequence-based reagent	Primers for *Bcl11a* (Rat): Forward: *GTGACACCAGAAGATGACGA* Reverse: *ATTAGGGCTCCGTGTGCAG*	This paper	NA	Amplicon: 131 bp
Sequence-based reagent	Primers for *Actb* (Rat): Forward: *CACCATGTACCCAGGCATTG* Reverse: *CACACAGAGTACTTGCGCTC*	This paper	NA	Amplicon: 111 bp
Sequence-based reagent	Primers for *Hprt* (Rat): Forward: *CAGACTTTGCTTTCCTTGGTCA* Reverse: *CCAACACTTCGAGAGGTCCT*	This paper	NA	Amplicon: 89 bp

### gSWITCH

[This includes section g.1 to g.5].

#### g.1 Usage

It is designed to handle gene-expression data from lineage progression or time series or any variable-dependent experiments. Experiment type: bulk RNA-seq, single-cell RNA-seq experiments flattened to pseudo-bulk. In the series data, the number of series or time points or group >3 and the replicates per time point or group >2.

#### g.2 Access

Freely available at https://altoslabs.shinyapps.io/gSWITCH/. gSWITCH is also available at the GitHub repository: https://github.com/Tanay-math/gSWITCH (Ghosh, 2026a). The repository contains the source code, installation instructions, a step-by-step tutorial, and example data. A containerised version is available at ghcr.io/tanay-math/gswitch:1.0.0, allowing the application to be run without separately installing R or the required R packages. For computationally intensive analyses, we recommend running gSWITCH locally either from the GitHub repository or using the containerised version.

#### g.3 Implementation

gSWITCH is a web application and operating system (OS) independent. gSWITCH is implemented in ‘R’ by using *reshape2*, *dplyr*, *DESeq2*, *ggplot2*, *tximport*, *MonoInc,* and *plotly* (R Development Core Team, 2019; Wickham, 2007; Wickham, 2016; Wickham, 2017; Love et al., 2014) and employing RStudio’s *shiny* and *shinycssloaders* packages for the web application.

#### g.4 Logic

gSWITCH is based on the following: (1) GLM (Generalised linear model) fit and hypothesis testing, (2) specific contrasts analysis, (3) absolute maximum/minimum (in a closed interval) and monotonicity to categorize switch types.

We refer the Figure 1—figure supplement 2. We employ DESeq2 for GLM fit, calculation of LTR (likelihood ratio test) p-value (adjusted by FDR) and select genes for which adjusted p-value (Adj p)<user-defined value (u.d.) [0.05 is set as default]. Let’s say these genes are our set (i). We then calculate the contrast between the first and the last cell state or time points and the genes for which Adj p<user defined (or 0.05 as default) and the magnitude of fold change |FC|>user-defined value (or 1.4 is set as default) was considered as set (ii). Intersection of (i) and (ii) was taken to identify four different switch types which are based on absolute max/min and monotonicity. A relative expression value (g) was calculated by determining expression level of each cell state relative to the mid-point [for n=odd number: mid-point={(n/2)+0.5}^th^ cell state; for n=even number: mid-point=(n/2)^th^ cell state].

1. Type 1: properties: (a) absolute maxima at the end point (b) ‘g’ is monotonically increasing i.e., within [g1, g_n_], g_j-1_ <g_j_ (j=1,2,…n) n is a positive integer >3 (c) contrast analysis: X_n_ vs X_n-1_: Adj *P*<u.d. (or 0.05), |FC|>1.4 (or u.d.).
2. Type 2: properties: (a) absolute maxima at the mid-point (b) ‘g’ is monotonically increasing from start to mid-point and then monotonically decreasing from the mid-point to end point i.e., within [g_1_,g_j_], g_j_>g_j-1_ AND [g_j_,g_n_], g_j_> g_j+1_ (c) contrast analyses: X_j_ vs X_j+1_ AND X_j-1_ vs X_j_: Adj *P*<0.05 (or u. d.), |FC|>1.4 (or u.d.).
3. Type 3: properties: (a) absolute maxima at the start point (b) ‘g’ is monotonically decreasing as it progresses to end point i.e., within [g_1_, g_n_], g_j-1_ >g_j_ (c) contrast analysis: X_1_ vs X_2_: Adj *P*<0.05 (or u.d.), |FC|>1.4 (or u.d.).
4. Type 4: properties: (a) absolute minima at the mid-point (b) ‘g’ is monotonically decreasing from start to the mid-point and then monotonically increasing from the mid-point to the end point i.e., within [g_1_,g_j_], g_j-1_ >g_j_ AND [g_j_,g_n_], g_j_< g_j+1_. (c) contrast analyses: X_j_ vs X_j+1_ AND X_j-1_ vs X_j_: Adj *P*<0.05 (or user defined), |FC|>1.4 (or u.d.).

##### Identifying transcription factors (TFs)

Comprehensive list of TFs were retrieved from the TFcheckpoint database (Chawla et al., 2013), an experimental evidence-based database. L∩S is the TFs in the switch genes. L: list from TFcheckpoint, S: switch genes.

### g.5 Parameters used in this paper

Adj *P*<0.05, FC = 1.5.

### RNAseq analysis

RNA was isolated using RNA MiniPrep (Zymo Research, D7001) following the manufacturer’s protocol. DNase treatment on column (as per manufacturer protocol) was included in this isolation protocol. Paired-end directional library was prepared using NuGen kit: Ovation RNA-seq Systems 1–16 for model organisms (0349–32).

Oligodendrocyte lineage dataset: The accession number of this dataset is GSE301578. Data were pre-processed through SortMeRNA and Trimmomatic. Rat transcriptome (Rnor_6.0.cdna+Rnor_6.0.ncrna) index was built and quantification was performed using Salmon (quasi-mode) (Patro et al., 2017). Data was normalised using DESeq2 (Love et al., 2014). This tximport of Salmon quantification was run on gSWITCH (accessible at https://altoslabs.shinyapps.io/gSWITCH/, https://github.com/Tanay-math/gSWITCH, Ghosh, 2026a) for switch gene identification and visualisation.

Analysis of aged and young Rat OPC data: The accession number of this dataset is GSE303317. Data were pre-processed using sortMeRNA (Kopylova et al., 2012) and Trimmomatic (Bolger et al., 2014). The rat transcriptome index (Rnor_6.0.cdna+Rnor_6.0.ncrna) was built, and quantification was performed using Salmon in quasi-mapping mode. Raw Salmon counts were imported using the tximport package. Differential expression analysis was conducted with DESeq2, incorporating batch as a covariate in the design formula: design=~batch + group. P-values were adjusted for multiple testing using the false discovery rate (FDR) method. Genes with an adjusted p-value less than 0.05 and a fold change greater than or equal to 1.5 were considered differentially expressed between the two groups. For data visualisation, counts were normalised using a variance-stabilizing transformation (VST) (varianceStabilizingTransformation function from DESeq2), and batch effects were removed using the removeBatchEffect function from the limma package (Ritchie et al., 2015).

### TF-binding site enrichment analysis

Using type 3 and type 4 switches as input, we employed the ChIP-X Enrichment Analysis (ChEA3) tool (https://maayanlab.cloud/chea3/) (Keenan et al., 2019) and utilised two experimentally curated ChIP-seq databases – ENCODE (Encyclopedia of DNA Elements) (ENCODE Project Consortium, 2012) and ReMap2 (Regulatory Map database) (Hammal et al., 2022) – to identify transcription factors (TFs) with significantly enriched binding sites (FDR <0.05). We then focused on transcription factors consistently identified by both ENCODE and ReMap2.

### GO (Gene ontology) analysis

DAVID version 6.8 (the Database for Annotation, Visualisation and Integrated Discovery) (Huang et al., 2009) functional annotation tool was used for GO terms overrepresentation analysis. Benjamini-Hochberg adjusted p-value <0.05 was considered significant.

### dN/dS analysis

Twenty-three vertebrate species (including lamprey), spanning a broad range of vertebrate phyla, were used in this study (Supplementary file 4). The longest (XL) coding sequences (CDS) and the corresponding protein sequences of *Bcl11a* were obtained from Ensembl (Dyer et al., 2025). For lamprey, the sequences were retrieved from GenBank (Sayers et al., 2021), as they were not available in Ensembl. Protein sequences were aligned using MAFFT (Katoh and Standley, 2013), and the resulting alignment together with the corresponding coding sequences was used as input to the PAL2NAL program (Suyama et al., 2006) to generate codon-based nucleotide alignments. The species tree was generated from CDS using the NGPhylogeny server (Lemoine et al., 2019), the nucleotide sequence alignment, BMGE (Criscuolo and Gribaldo, 2010) for alignment curation, and PhyML (Guindon et al., 2010) for tree inference. dN/dS ratios and likelihood ratio tests of model comparisons were performed using Codeml in the PAML package v4.8a (Yang, 2007). Codon frequency was set to F3X4, the universal genetic code was specified in the icode variable, and the program was run with the following selection model parameters: Nssites 0 and 3 (selection models M0 and M3), Nssites 1 and 2 (models M1a and M2a), and Nssites 7 and 8 (models M7 and M8). Significance of log-likelihood differences for model comparisons was tested using χ^2^ statistics with appropriate degrees of freedom, as described in the PAML manual (Yang, 2026).

Further detail on the M7 and M8 comparison: these models differ only by M8 allowing an additional class of sites with dN/dS >1. The likelihood ratio test (LRT) comparing these indicates a significant preference for M8 (p=0.006) but identifies only a single site with posterior mean dN/dS >1 and confirms all other sites to be evolving under purifying selection. (Codon 156, i.e., nucleotide positions 466–468 in our curated alignment, is inferred to have dN/dS=2.93±2.81 and posterior probability 0.94 of being under positive selection. However, inspection of the alignment reveals that this position is represented only in macaque, rabbit, blue tit, and frog, making evidence for positive selection across species unconvincing even at this one site.)

### Animals

Sprague–Dawley rats (C. River, Margate, UK) and C57Bl/6 mice (18-month-old) were housed in individually ventilated cages at 22 ±1 °C and 60±5% humidity under a 12 hr light–dark cycle. Food and water were provided ad libitum in a standard rodent facility at the University of Cambridge, UK or Altos Labs-Cambridge Institute of Science, UK. All procedures were carried out under the Home Office Project Licence PC0C0F291 in accordance with the Animals (Scientific Procedures) Act 1986 (Amendment Regulations 2012) and were approved by the Animal Welfare and Ethical Review Body (AWERB) of the University of Cambridge and Agenda Resource Management. The study followed the ARRIVE guidelines.

### Cellular isolation

Rats were sacrificed using an overdose of pentobarbital and the whole brain was dissected immediately in ice cold Hibernate A low fluorescence (HALF) medium (PMID: 31585093). Subsequently, cellular isolation was performed as described. Per 10 million cell suspension, we used 2.5 μg of primary antibody. Oligodendrocytes (OL) were isolated first using goat-anti-mouse-MOG-Biotinylated (R&D systems, BAF2439); followed by OPC were isolated from the MOG negative population using mouse-anti-rat-A2B5-IgM antibody (Millipore; MAB312). Finally, pre-oligodendrocytes (PreOL) were isolated from MOG and A2B5-negative population using mouse anti-O4-IgM (R&D Systems, MAB1326). The respective secondary antibodies (20 μl) were: mouse-anti-biotin magnetic micro beads (Miltenyi, 130-105-637), rat-anti-mouse-IgM antibody magnetic beads (Miltenyi, 130-047-302), rat-anti-mouse-IgM magnetic beads (Miltenyi; cat#130-047-302).

### Cell culture and differentiation

Culture of aged OPCs (isolated from 20 to 24 month-old rats) was performed as described (PMID: 31585093). Additionally, DMEM-F12 (Sigma), GlutaMAX (Gibco), and B27 supplement (Gibco) were used in the media for aged OPC culture. Proliferation medium contains PDGF and bFGF at 30 ng/ml each (PeproTech). For differentiation as described (Ghosh et al., 2024), OPC medium was supplemented with 40 ng ml^–1^ 3,3',5-Triiodo-L-thyronine (T3) (Sigma; T6397). Medium was replaced on alternate days for 5 days.

Neonatal OPCs (isolated from P7 rats) were cultured as described (Ghosh et al., 2024).

### Cloning of Bcl11a

The original Human XL *BCL11A* clone was a gift from Prof. Walid Khaled lab (Cambridge University, UK). *BCL11A* was subcloned into a TOPO vector (pCR 2.1-TOPO TA using TOPO TA Cloning Kit following manufacturer instructions) and the sequence was verified. For in vitro transcription, the following primers were used for PCR amplification of *BCL11A*:

FP: 5′ ***TAATACGACTCACTATAG***gccaccATGTCTCGCCGCAAGCAA 3′

(bold: T7 promoter sequence, small case: Kozac sequence)

RP: 5′ *CTATTCAGTTTTTATATCATTATTCAACACTCGATCA* 3′

Purified PCR product (from above) was used as a template and transcribed in vitro using HiScribe T7 ARCA mRNA Kit (New England Biolabs) with tailing and modified nucleotides (Tebu Bioscience) following the manufacturer’s protocol (E2060S). mRNA was purified using Directzol RNA Miniprep kit (Zymo Research). CleanCap^R^ EGFP mRNA (L-7601) was procured from Tebu Bioscience.

### Western blot analysis

MCF7 cells were used to verify protein-level expression of BCL11A following transfection with in vitro–transcribed *Bcl11a* mRNA (see above). MCF7 cells were selected because they exhibit poor endogenous BCL11A expression (Waterhouse M et al., 2025). MCF7 cells tested negative for mycoplasma contamination. For the mRNA transfection method (see below). After 36 hr of transfection, cells were lysed in the wells using RIPA buffer containing protease inhibitors. The lysates were incubated at 4 °C for 30 min and centrifuged at maximum speed at 4 °C for 30 min. The resulting supernatant was used for protein separation on a 7.5% SDS–PAGE gel and subsequently transferred to a PVDF membrane for Western blotting. Antibodies used were, anti-BCL11A (ab191401, Abcam, 1:3000) (Waterhouse et al., 2025) and an HRP-conjugated donkey anti-rabbit IgG secondary (NA934, Amersham/GE Healthcare) (1:10,000); anti-beta Actin antibody (ab6276) (1:3000) and HRP-conjugated sheep anti-mouse IgG secondary (NA931, Amersham/GE Healthcare) (1:10,000). Immunoreactive bands were detected using ECL reagent (Amersham) and imaged an Odyssey Imaging system.

### Transfection

siRNA transfection in neonatal OPCs: We used Lipofectamine RNAiMAX transfection reagent (ThermoFisher scientific) and followed the protocol as described (Ghosh et al., 2024). siRNAs were procured from Dharmacon. For *Bcl11a* we used siGENOME Rat *Bcl11a* (305589)-SMARTpool (Cat no. M-085668-01-0010); for control we used siGENOME non-Targeting siRNA Pool #2 (Cat no. D-001206-14-05).

mRNA transfection in aged OPCs: 60,000 cells per well of a 24-well plate were seeded. Cells were kept in proliferation media [PDGF and bFGF, 30 ng/ml each (Peprotech)] for 5 days. mRNA (approx. 500 ng) was transfected using Lipofectamine MessengerMax transfection reagent (Thermo Fisher scientific) following the manufacturer’s protocol.

### Immunofluorescence

Cells were fixed in 4% paraformaldehyde (PFA) for 10 min at room temperature, washed in PBS, and processed for immunofluorescence staining as described previously (Neumann et al., 2019). For in vivo experiments, animals were perfused with 4% PFA in PBS; spinal cords were post-fixed overnight in 4% PFA, cryoprotected in 20% sucrose, and embedded in OCT medium (Tissue-Tek). Immunofluorescence staining was performed on 12 µm cryostat sections following the previously described method (Rawji et al., 2018).

The antibodies used in this study, described previously (Ghosh et al., 2024), were: rat anti-MBP (MCA409S, Serotec) [1:500], rabbit anti-OLIG2 (AB9610, Millipore) [1:1000 or 1:100], chicken anti-GFP (ab13970, Abcam) [1:1000], donkey Cy3 AffiniPure anti-Rat (712-165-150, Jackson Immuno Research) [1:1000], donkey Alexa Fluor 647 anti-Rabbit (A31573, Invitrogen) [1:1000 or 1:500], donkey Alexa Fluor 488 Anti-Chicken (703-546-155, Jackson Immuno Research) [1:1000].

Images were acquired using an SP8 (Leica TCS) confocal microscope. All acquisition settings were kept constant throughout the experiment. Images were processed in ImageJ version 2.1.0 (Schindelin et al., 2012). Cell counting was performed using the Cell Counter tool in ImageJ. Immunofluorescence experiments were performed and analysed blinded to treatment.

### EdU proliferation assay

For EdU incorporation, cells were incubated with 10 µM EdU for 5 hr and subsequently processed following the manufacturer’s instructions (Click-iT Plus EdU Alexa Fluor 647 Imaging Kit, Thermo Fisher, C10640). In vitro tissue-culture cells were fixed in 4% paraformaldehyde (Thermo Fisher, 10131580) for 10 min at room temperature. Cells were washed once with PBS and blocked in PBS containing 0.1% Triton X-100 and 5% donkey serum (Sigma, D9663) for 30 min at room temperature. EdU detection was performed according to the kit protocol, followed by immunostaining. Antibodies used were: Anti-OLIG2: rabbit anti-OLIG2 (AB9610, Millipore) [1:1000], Goat Alexa Fluor 488 anti-rabbit IgG (A11008, Invitrogen) (1:1000) [siRNA experiments in Figure 4], Goat Alexa Fluor 594 anti-rabbit IgG (A11012, Invitrogen) (1:1000) [mRNA transfection experiment in Figure 5].

### RT-qPCR

Total RNA was isolated using the mirVana kit (Ambion). RNA samples were treated with DNase I (Thermo Fisher Scientific) prior to reverse transcription. cDNA was generated using random hexamers (Invitrogen) and SuperScript IV Reverse Transcriptase (Invitrogen) according to the manufacturer’s protocol. Real-time PCR was performed using SYBR Green Master Mix (Applied Biosystems) on a QuantStudio 7 Flex system (Applied Biosystems). The PCR program was: 50 °C for 2 min, 95 °C for 10 min, followed by 40 cycles of 95 °C for 15 s and 60 °C for 1 min. A melt-curve (dissociation) analysis was performed at the end.

Relative quantification of qPCR data was performed using the Pfaffl method (Pfaffl, 2001). PCR efficiencies for the target gene (*Bcl11a*) and the endogenous control (*Actb*) were determined from the slopes of their respective standard curves. For qPCR performed and presented in Figure 3—figure supplement 1, *Hprt* was used as an endogenous control for normalisation.

Rat-specific primers:

Bcl11a:

F: *GTGACACCAGAAGATGACGA*

R: *ATTAGGGCTCCGTGTGCAG*

[Amplicon: 131 bp]

Actb:

F: *CACCATGTACCCAGGCATTG*

R: *CACACAGAGTACTTGCGCTC*

[Amplicon: 111 bp]

Hprt:

F: *CAGACTTTGCTTTCCTTGGTCA*

R: *CCAACACTTCGAGAGGTCCT*

[Amplicon: 89 bp]

Primer sequences for *Pdgfra* and *Mbp* were as described previously in Ghosh et al., 2024.

### AAV delivery

The *Sox10* multiple-species conserved enhancer element 5, conjugated to the *cfos* basal promoter (Addgene #115783; Pol et al., 2013), was used to drive oligodendrocyte-lineage–specific expression of *Bcl11a* and *EGfp* (Figure 6A). For the control construct, the same regulatory sequence drove expression of *EGfp* alone. The entire *Sox10*-driven expression cassette (Figure 6A) was cloned between the inverted terminal repeats (ITRs) of an adeno-associated virus 2 (AAV2) vector. Cloning, large-scale AAV-PHP.eB packaging, and purification were performed by GenScript (UK).

AAV carrying the construct (Figure 6A) was administered to 18-month-old mice via tail vein injection at a dose of 5×10¹¹ genome copies (GC) in a total volume of 200 µL.

### Focal demyelination of the spinal cord

A mouse spinal cord demyelination model was generated by injecting 1% lysophosphatidylcholine (lysolecithin, LPC; Sigma-Aldrich, Gillingham, UK) prepared in PBS into the ventral white matter of the spinal cord at the Th13/L1 level following surgical exposure under isoflurane anaesthesia. 18-month-old C57Bl/6 mice were used in this study; the animals do not manifest abnormalities in this model. Mice were sacrificed by perfusion fixation at 14 days post lesioning (dpl) for histological analysis.

### *Plp1* in situ hybridisations (ISH)

We followed standard procedures used in the Richardson laboratory (UCL) (https://www.homepages.ucl.ac.uk/~ucbzwdr/In%20Situ%20Protocol.pdf). *Plp1* mRNA was detected using a digoxigenin (DIG)-labelled riboprobe. Primers (FP: *GGGGATGCCTGAGAAGGT*; RP: *TGTGATGCTTTCTGCCCA*) targeting the Allen Brain Atlas Plp1 sequence (ABA experiment 75496148) were used to amplify the region, which was subsequently TA-cloned into the pCRII vector using the Thermo Fisher K206001 kit. Insert orientation was confirmed by Sanger sequencing. Following linearisation with XbaI, an antisense DIG-labelled riboprobe (970 bp) was synthesised by in vitro transcription using T7 RNA polymerase.

For hybridisation, the DIG-labelled probe was diluted 1:1000 in hybridisation buffer [50% de-ionised formamide, 1× salts (0.2 M NaCl, 5 mM EDTA, 10 mM Tris-HCl pH 7.5, 5 mM NaH₂PO₄, 5 mM Na₂HPO₄), 0.1 mg/ml yeast tRNA, 10% dextran sulphate, and 1× Denhardt’s solution], denatured at 70–75 °C for 10 min, and applied to tissue sections. Slides were incubated overnight at 65 °C in a humidified chamber containing 50% formamide/1 X SSC. The next day, sections were washed in three pre-warmed solutions of 50% formamide/1 X SSC/0.1% Tween-20 at 65 °C, followed by two room-temperature washes in 1 X MABT (100 mM maleic acid, 150 mM NaCl, 0.1% Tween-20, pH 7.5). Sections were blocked for 1 h in 20% sheep serum and 5 X blocking reagent (2% Roche blocking reagent+10% heat-inactivated sheep serum) in 1 X MABT, then incubated overnight at 4 °C with anti-DIG-AP antibody (1:1000) prepared in the same blocking solution. After three washes in MABT, colour development was performed using NBT/BCIP in a freshly prepared Tris/MgCl_₂_/NaCl developing buffer with 5% polyvinyl alcohol (PVA) at pH 9.8, incubated at 37 °C in the dark until optimal signal intensity was achieved. Slides were rinsed in PBS, counterstained with DAPI, and mounted in Fluoromount (Sigma) and imaged under Thermo Fisher EVOS M5000 Cell Imaging System. ISH experiments were performed and analysed blinded to treatment.

### Statistical analysis

R (version 4.0.2) or GraphPad Prism (version 8.4.3) was used for statistical analyses. Normality was evaluated using the Shapiro–Wilk test, and Welch’s t-test was used in cases of heteroscedasticity. Sample sizes were determined by power analysis, assuming a power of 0.8 and a level of significance of 0.05, with the expected effect size and variability estimated from comparable experiments previously conducted in our laboratory. Both male and female animals were included without selection by sex. Sex was not analysed as an experimental factor. Animals were randomly allocated to groups. Data affected by accidental technical failures were excluded; no other exclusions were made.

## Data Availability

RNA-seq data have been deposited in GEO under accession numbers GSE301578 and GSE303317. gSWITCH is accessible here: https://github.com/Tanay-math/gSWITCH, copy archived at Ghosh, 2026a. Codes are deposited in the GiHub: https://github.com/Tanay-math/BCL11A-oligodendrocyte-lineage-ageing, copy archived at Ghosh, 2026b. The following datasets were generated: BarorR GhoshT FranklinRJM 2026Gene expression analysis in oligodendrocyte lineageNCBI Gene Expression OmnibusGSE301578 GhoshT 2025Analysis of aged and young Rat OPC data (bulk RNA-seq; Re-analysis)NCBI Gene Expression OmnibusGSE303317

## Additional files

Supplementary file 1. All switches during oligodendrocyte (OL)-lineage progression.

Supplementary file 2. Transcription factor (TF) switches during OL-lineage progression.

Supplementary file 3. 27 Transcription factors (TFs) identified by both Encode and Remap2.

Supplementary file 4. *Bcl11a* DNA and protein sequence accession numbers for 23 vertebrate species used in this study.

MDAR checklist

## Appendix 1

Please see Figure 1—figure supplement 1. We observed elevated expression of *Hes1* in OPCs compared with both PreOLs and OLs, consistent with its role as a Notch effector that maintains the OPC progenitor state and inhibits oligodendrocyte maturation (Brosnan and John, 2009; Ogata et al., 2011).

Compared with PreOLs, adult OPCs isolated from 2 to 3 month-old rats did not show higher RNA expression of canonical OPC markers: *Pdgfra*, *Pcdh15*, and *C1ql1*. However, as expected, OPCs expressed higher levels of these markers than mature OLs. One possible explanation is the intrinsic heterogeneity of adult OPC populations. Adult OPCs exist in multiple transcriptional states, including quiescent-like and differentiation-primed states. During early differentiation, OPC markers, such as *Pdgfra* may not be immediately extinguished, and PreOLs may transiently retain these transcripts. The PreOL population captured in our study represents intermediate states transitioning from OPCs to OLs, potentially still carrying residual OPC-associated RNAs from activated OPCs. Therefore, comparison of PreOLs with the total heterogeneous OPC pool, which includes quiescent-like OPCs, may give the appearance of higher canonical OPC marker expression in PreOLs.

Among the PreOL-specific markers, *Gpr17* clearly distinguished the PreOL state in our data, showing higher expression compared with both OPCs and OLs. *Bcas1* and *Enpp6* showed higher expression in PreOLs compared with OPCs. However, when PreOLs were compared with OLs, *Bcas1* appeared lower in PreOLs, whereas *Enpp6* expression remained largely unchanged.

The OL markers *Mobp* and *Mog* showed significantly higher expression in OLs compared with OPCs, whereas their expression was not altered between OPCs and PreOLs. In contrast, the canonical OL maturity marker *Mbp* showed a progressive and significant increase during differentiation, with expression levels clearly following the expected pattern: OL>PreOL> OPC.

We did not detect any difference in the astrocyte marker *Gfap* across these cell-type comparisons, suggesting a similar level of unavoidable astrocytic contamination across groups. Therefore, such contamination is unlikely to confound the interpretation of differential gene expression among OPCs, PreOLs and OLs.
