# Supplementary material for: The transcription factor BCL11A restores differentiation potential to aged oligodendrocyte progenitor cells
Source: eLife. 2026 Oct 26;15:RP110038. doi: 10.7554/eLife.110038 (PMC13707380; doi:10.7554/eLife.110038)
Supplement: Supplementary file 2 [file elife-110038-supp3.docx]

**Supplementary File 3.** 27 Transcription factors (TFs) identified by both Encode and Remap2.

BATF

**BCL11A**

BHLHE40

**CEBPB**

ETS1

FOS

**FOXM1**

GTF2B

IKZF1

**JUN**

JUND

MAFF

MEF2A

MYB

NFE2

NFIC

PAX5

**PRDM1**

RELA

SPI1

STAT1

STAT3

TAL1

TCF12

TCF3

TEAD4

ZNF384

FDR<0.05. Bold: Five TFs present in Type 3 switches.
