# Supplementary material for: The transcription factor BCL11A restores differentiation potential to aged oligodendrocyte progenitor cells
Source: eLife. 2026 Oct 26;15:RP110038. doi: 10.7554/eLife.110038 (PMC13707380; doi:10.7554/eLife.110038)
Supplement: Supplementary file 3 [file elife-110038-supp4.docx]

| **Species** | **cds accession** | **Protein accession** |
| --- | --- | --- |
| Human | ENST00000642384.2 | ENSP00000496168 |
| Mouse | ENSMUST00000109514.8 | ENSMUSP00000105140 |
| Rat | ENSRNOT00000106316.1 | ENSRNOP00000093688 |
| Chimpanzee | ENSPTRT00000046502.3 | ENSPTRP00000042480 |
| Gorilla | ENSGGOT00000067107.1 | ENSGGOP00000038984 |
| Elephant | ENSLAFT00000003101.3 | ENSLAFP00000002578 |
| Horse | ENSECAT00000132103.1 | ENSECAP00000085653 |
| Megabat | ENSPVAT00000000041.1 | ENSPVAP00000000040 |
| Macaque | ENSMMUT00000097395.1 | ENSMMUP00000074838 |
| Rabbit | ENSOCUT00000001202.4 | ENSOCUP00000001042 |
| Chicken | ENSGALT00010041405.1 | ENSGALP00010024257 |
| Turkey | ENSMGAT00000014673.3 | ENSMGAP00000013758 |
| Blue-tit | ENSCCET00000017097.1 | ENSCCEP00000010895 |
| Painted turtle | ENSCPBT00000044255.1 | ENSCPBP00000037734 |
| Lizard | ENSPMRT00000007787.1 | ENSPMRP00000007275 |
| *Xenopus tropicalis* | ENSXETT00000097709.2 | ENSXETP00000091923 |
| Zebrafish | ENSDART00000087426.7 | ENSDARP00000081860 |
| Elephant shark | ENSCMIT00000005454.1 | ENSCMIP00000005270 |
| Stickleback | ENSGACT00000017770.1 | ENSGACP00000017735 |
| Atlantic_cod | ENSGMOT00000072043.1 | ENSGMOP00000061821 |
| Opossum | ENSMODT00000074969.1 | ENSMODP00000042447 |
| Coelacanth | ENSLACT00000004033.1 | ENSLACP00000003997 |
| Lamprey | XM_032948470.1 | XP_032804361.1 |

**Supplementary File 4**. *Bcl11a* DNA and protein sequence accession numbers for 23 vertebrate species used in this study.
