# Supplementary material for: The transcription factor BCL11A restores differentiation potential to aged oligodendrocyte progenitor cells
Source: eLife. 2026 Oct 26;15:RP110038. doi: 10.7554/eLife.110038 (PMC13707380; doi:10.7554/eLife.110038)
Supplement: Peer review history

# Editor report: eLife Assessment

**DOI:** 10.7554/eLife.110038.3.sa0

**Authors:** Samuel Pleasure

**Affiliations:** University of California, San Francisco United States

**Keywords:** Convincing, Valuable

This **valuable** study demonstrates molecular changes associated with age related impairment in oligodendrocyte differentiation and ability to myelinate. The identification of particular genes that are associated with this decline will provide potential future targets for therapeutic interventions. The reviewers felt that the quality of the evidence was **convincing** while identifying some minor weaknesses that were largely addressed in the review process.

# Referee report: Reviewer #1 (Public review)

**DOI:** 10.7554/eLife.110038.3.sa1

Summary:

This manuscript by Ghosh and colleagues investigates the transcriptional changes within the oligodendrocyte lineage that contribute to age-related declines in oligodendrocyte differentiation and myelination. Combining bulk RNA-Seq on acutely purified oligodendrocyte lineage cells with bioinformatic approaches, the authors identify groups of genes that show different patterns of dynamic regulation during differentiation (which they term "switch" genes, or "switches"). A subset of these switch genes are differentially regulated with age. The authors identify two transcription factors, Bcl11a and Foxm1 that are downregulated during differentiation, have predicted binding site enrichment at other switch genes and are downregulated in aged OPCs. Functionally testing Bcl11a, the authors show that Bcl11a knockdown inhibits the differentiation of young OPCs in culture, whereas overexpression promotes differentiation of aged OPCs. Viral expression of Bcl11a in Sox10 expressing cells accelerates the formation of Plp1+ oligodendrocytes in aged rodents following lysolecithin induced demyelination.

Strengths:

The work is clearly presented and addresses an important biological problem. The bioinformatic approaches used in the manuscript are powerful, and the identification of Bcl11a as a modulator of oligodendrocyte differentiation is a novel finding. The combined in vitro and in vivo approaches to assess the function of Bcl11a in oligodendrocyte differentiation are a substantial strength of the work.

Comment on revised version.

In the revised version the authors now provide analysis of expression of stage-specific markers for OPCs, preOls and OLs in their isolated cells. It is slightly concerning that the OPC markers show higher expression in the isolated preOLs than in the isolated OPCs, but the authors do provide some discussion on this point in the supplementary text.

# Referee report: Reviewer #2 (Public review)

**DOI:** 10.7554/eLife.110038.3.sa2

Ageing poses a significant challenge to the regenerative capacity of oligodendrocyte precursor cells (OPCs). Myelin abnormalities accumulate with age, while the ability of OPCs to differentiate into myelinating oligodendrocytes progressively declines. This likely contributes to inefficient replacement of damaged myelin and oligodendrocytes, impaired remyelination following injury, and reduced adaptive myelination. Identifying the molecular changes associated with this decline is therefore important for understanding and potentially treating age-related deterioration of CNS white matter.

This study sought to identify transcriptional regulators involved in oligodendrocyte-lineage progression whose expression is altered in aged OPCs. The authors developed gSWITCH, a computational tool that identifies genes showing defined dynamic expression patterns across ordered biological states. By combining this analysis with comparisons of young and aged OPC transcriptomes and transcription-factor-binding-site enrichment, they identified Bcl11a as a candidate regulator. Bcl11a transcripts are abundant in young OPCs, decline during oligodendrocyte differentiation, and are markedly reduced in aged OPCs.

A major strength of the study is its combination of computational candidate identification with functional experiments. Bcl11a knockdown substantially impaired the differentiation of young OPCs without measurably affecting their proliferation. Conversely, transient Bcl11a overexpression increased the differentiation of aged OPCs in vitro. Oligodendrocyte-lineage-specific expression of Bcl11a in aged mice also increased the generation of PLP1-positive oligodendrocytes following focal demyelinating injury. Together, these complementary loss- and gain-of-function experiments support the conclusion that Bcl11a expression is functionally important for OPC differentiation and that restoring its expression can improve the differentiation competence of aged OPCs.

While the transcription-factor-binding-site enrichment analysis predicts a Bcl11a-regulated network, the current study does not establish direct binding or identify the downstream genes responsible for its effect on OPC differentiation. Similarly, the upstream mechanisms responsible for the age-associated reduction in Bcl11a expression were not investigated. Further work may help establish a more complete mechanistic framework explaining how restoration of Bcl11a expression improves OPC differentiation.

Overall, this study offers valuable insights into the age-related loss of regenerative capacity in the central nervous system and introduces a computational framework that may be broadly useful for investigating dynamic gene regulation in other biological contexts.

Comments on revised version.

The authors have addressed my previous comments, and the revised manuscript has been substantially strengthened by the inclusion of additional supporting data and an expanded discussion.

# Author comment: Author response

**DOI:** 10.7554/eLife.110038.3.sa3

**Authors:** Tanay Ghosh, Roey Baror, Chao Zhao, Amar Sharma, Wing Hei Au, András Lakatos, Nick Goldman, Robin JM Franklin

**Affiliations:** Altos Labs-Cambridge Institute of Science, Cambridge CB21 6GP, United Kingdom Cambridge United Kingdom; Wellcome-MRC Cambridge Stem Cell Institute, University of Cambridge, Cambridge, CB2 0AW, United Kingdom Cambridge United Kingdom; Altos Labs-Cambridge Institute of Science, Cambridge CB21 6GP, United Kingdom Cambridge United Kingdom; University of Cambridge Cambridge United Kingdom; John van Geest Centre for Brain Repair, Department of Clinical Neurosciences, University of Cambridge, Cambridge, CB2 0PY, UK Cambridge United Kingdom; John van Geest Centre for Brain Repair, Department of Clinical Neurosciences, University of Cambridge, Cambridge, CB2 0PY, UK Cambridge United Kingdom; EMBL-European Bioinformatics Institute Hinxton United Kingdom; Altos Labs Cambridge United Kingdom

The following is the authors’ response to the original reviews.

**Public Reviews:**

**Reviewer #1 (Public review):**

Summary:

This manuscript by Ghosh and colleagues investigates the transcriptional changes within the oligodendrocyte lineage that contribute to age-related declines in oligodendrocyte differentiation and myelination. Combining bulk RNA-Seq on acutely purified oligodendrocyte lineage cells with bioinformatic approaches, the authors identify groups of genes that show different patterns of dynamic regulation during differentiation (which they term "switch" genes, or "switches"). A subset of these switch genes is differentially regulated with age. The authors identify two transcription factors, Bcl11a and Foxm1, that are downregulated during differentiation, have predicted binding site enrichment at other switch genes, and are downregulated in aged OPCs. Functionally testing Bcl11a, the authors show that Bcl11a knockdown inhibits the differentiation of young OPCs in culture, whereas overexpression promotes the differentiation of aged OPCs. Viral expression of Bcl11a in Sox10-expressing cells accelerates the formation of Plp1+ oligodendrocytes in aged rodents following lysolecithin induced demyelination.

Strengths:

The work is clearly presented and addresses an important biological problem. The bioinformatic approaches used in the manuscript are powerful, and the identification of Bcl11a as a modulator of oligodendrocyte differentiation is a novel finding. The combined in vitro and in vivo approaches to assess the function of Bcl11a in oligodendrocyte differentiation are a substantial strength of the work.

We sincerely thank the reviewer for their positive assessment and for recognising the significance of our study, as well as the bioinformatics approach and tool developed as part of this work.

Weaknesses:

Although the PCA plots show distinct and reproducible global gene expression differences between the different isolated cell populations, the authors do not present a figure showing expression levels of typical stage-specific markers (e.g., Pdgfra, Pcdh15, C1ql1 for OPCs, Bcas1, Enpp6, Gpr17 for preOLs, Mobp, Mog, etc. for OLs) or confirm the absence of markers of other lineages (astrocytes, neurons, microglia, etc.). This makes it difficult to evaluate the success of their cell isolation strategy at different ages without reanalyzing the raw data.

Thank you for this suggestion. We have presented markers expression in a new figure (Supplementary Figure 1) and included a description in the new Supplementary text.

We observed elevated expression of Hes1 in OPCs as compared to both PreOL and OL, consistent with its role as a Notch effector that maintains the OPC progenitor state and inhibits oligodendrocyte maturation (PMID: 19104146, PMID: 21167918).

Compared with PreOLs, adult OPCs isolated from 2–3-month-old rats did not show higher RNA expression of canonical OPC markers: Pdgfra, Pcdh15, and C1ql1. However, as expected, OPCs expressed higher levels of these markers than mature OLs.

One possible explanation is the intrinsic heterogeneity of adult OPC populations. Adult OPCs exist in multiple transcriptional states, including quiescent-like and differentiation-primed states. During early differentiation, OPC markers such as Pdgfra are not immediately extinguished, and PreOLs may transiently retain these transcripts. The PreOL population captured in our study represents intermediate states transitioning from OPC to OL, potentially still carrying residual OPC-associated RNAs from activated OPCs. Therefore, comparing PreOLs with the total heterogeneous OPC pool, which includes quiescent-like OPCs, may give the appearance of higher canonical OPC marker expression in PreOLs.

Among the PreOL-specific markers, Gpr17 clearly distinguished the PreOL state in our data, showing higher expression compared with both OPCs and OLs. Bcas1 and Enpp6 showed higher expression in PreOLs compared with OPCs. However, when PreOLs were compared with OLs, *Bcas1* appeared to be lower in PreOLs, whereas Enpp6 expression remained largely unchanged.

The OL markers Mobp and Mog showed significantly higher expression in OLs compared with OPCs, whereas their expression was not altered between OPCs and PreOLs. However, the canonical OL maturity marker Mbp showed a progressive and significant increase during differentiation, with expression levels clearly following the expected pattern OL > PreOL > OPC.

We did not find any difference of astrocytes marker Gfap in those cell types comparison, suggesting similar level of unavoidable contamination which will not affect determination of differential gene expression. Regarding this please also see reviewer #2 major point 1.

We now included this in the supplementary text:

“Please see Supplementary Figure 1. We observed elevated expression of Hes1 in OPCs compared with both PreOLs and OLs, consistent with its role as a Notch effector that maintains the OPC progenitor state and inhibits oligodendrocyte maturation (Brosnan et al, 2009; Ogata et al., 2011).

Compared with PreOLs, adult OPCs isolated from 2–3-month-old rats did not show higher RNA expression of canonical OPC markers: Pdgfra, Pcdh15, and C1ql1. However, as expected, OPCs expressed higher levels of these markers than mature OLs. One possible explanation is the intrinsic heterogeneity of adult OPC populations. Adult OPCs exist in multiple transcriptional states, including quiescent-like and differentiation-primed states. During early differentiation, OPC markers such as Pdgfra may not be immediately extinguished, and PreOLs may transiently retain these transcripts. The PreOL population captured in our study represents intermediate states transitioning from OPCs to OLs, potentially still carrying residual OPC-associated RNAs from activated OPCs. Therefore, comparison of PreOLs with the total heterogeneous OPC pool, which includes quiescent-like OPCs, may give the appearance of higher canonical OPC marker expression in PreOLs.

Among the PreOL-specific markers, Gpr17 clearly distinguished the PreOL state in our data, showing higher expression compared with both OPCs and OLs. Bcas1 and Enpp6 showed higher expression in PreOLs compared with OPCs. However, when PreOLs were compared with OLs, Bcas1 appeared lower in PreOLs, whereas Enpp6 expression remained largely unchanged.

The OL markers Mobp and Mog showed significantly higher expression in OLs compared with OPCs, whereas their expression was not altered between OPCs and PreOLs. In contrast, the canonical OL maturity marker Mbp showed a progressive and significant increase during differentiation, with expression levels clearly following the expected pattern: OL > PreOL > OPC.

We did not detect any difference in the astrocyte marker Gfap across these cell-type comparisons, suggesting a similar level of unavoidable astrocytic contamination across groups. Therefore, such contamination is unlikely to confound the interpretation of differential gene expression among OPCs, PreOLs and OLs.”

In the main text we have added the following text:

“The expression patterns of cell-type-specific markers were consistent with their being distinct OPC, Pre-OL, and OL populations (Supplementary Figure 1, see Supplementary text for detailed description).”

Please note that a detailed discussion of marker expression in the main text will disrupt the flow of the manuscript in manner we feel would detract from its clarity. We have therefore provided this discussion in the Supplementary Text.

In addition, other publicly available datasets (e.g., the Barres lab bulk RNA-Seq datasets from PMID 25186741 or the Castelo-Branco lab single cell datasets from PMID 27284195) do not show downregulation of Bcl11a during OL differentiation as is described here - this apparent discrepancy is not discussed.

Thank you for raising this point. We have now included new data as a Supplementary Figure 4. We performed RT-qPCR (reverse transcription followed by qPCR) to quantify Bcl11a expression and found that it was significantly lower in OLs than in OPCs, and significantly lower in aged OPCs than in young OPCs. These data were presented together with stage-specific markers.

Regarding the comparison with PMID: 25186741: we extracted Bcl11a FPKM values from their dataset (GSE52564) and plotted, as shown in Author response image 1. We found that Bcl11a expression is downregulated during differentiation. However, the dataset contains only two replicates, and the SEM between the two OL replicates is very high, which may have contributed to the apparent lack of clarity. With such high SEM and only two replicates, the statistical power is poor, making robust statistical inference difficult.

**Author response image 1.** Plotting of FPKM values of Bcl11a (obtained from GSE52564). mean + SEM shown along with individual data points. OPC: Oligodendrocytes progenitor cells, NFO: Newly formed oligodendrocytes, MO: myelinating oligodendrocytes. Dotted red line: linear regression line.

Regarding comparison with PMID 27284195: we contacted the Castelo-Branco laboratory, and they kindly provided us with the analysis shown below in Author response table 1. This analysis showed that Bcl11a expression is lower in myelinating oligodendrocytes (MOLs) compared with OPCs. The apparent discrepancy observed in the web interface is likely because MOLs are displayed separately by subtype in the online resource. In single-cell datasets, particularly earlier pre-10x datasets with relatively lower cell numbers and sparser transcript detection, visualisations such as violin plots or t-SNE plots can be difficult to interpret when expression is distributed across multiple subclusters. Therefore, directly examining the differential expression statistics, including fold-change and significance values, provides a clearer and more quantitative assessment of the expression change.

**Author response table 1.** Bcl11a expression difference in MOLs vs OPCs (dataset: GSE75330).

Gene	Comparison	avg_ log_(2)FC	p_val_adj	regulation
Bcl11a	MOLs vs OPCs	-1.57156	5.55E-07	down in MOLs

FC: fold change, p_val_adj: adjusted p-value.

Therefore, our bulk RNA-seq and RT-qPCR analyses presented in this paper are consistent with the Barres laboratory bulk RNA-seq dataset (PMID: 25186741) and the Castelo-Branco laboratory scRNAseq dataset (PMID: 27284195).

**Reviewer #2 (Public review):**

Aging poses a significant challenge to the regenerative capacity of oligodendrocyte precursor cells (OPCs) to differentiate and myelinate neuronal axons. Myelin abnormalities accumulate with age, and it is likely that the ability of OPCs to differentiate into myelinating oligodendrocytes becomes progressively impaired during aging, leading to inefficient turnover of damaged myelin and oligodendrocytes, as well as reduced adaptive myelination. Understanding the molecular mechanisms underlying the compromised capacity of aged OPCs is therefore critical for addressing age-related white matter decline.

This study aims to decipher the intrinsic molecular changes that occur in aged OPCs. By profiling differentially expressed transcription factors (TFs) between young and aged OPCs, and by employing a novel bioinformatic tool to identify key TFs that undergo dynamic changes across distinct stages of OPC differentiation, the authors identify Bcl11a as a potential regulator. Bcl11a is highly expressed in young OPCs but markedly reduced in aged cells. Functional experiments further demonstrate that while Bcl11a does not affect OPC proliferation, it significantly promotes the differentiation of aged OPCs. Importantly, this effect is also observed in vivo following demyelinating injury in aged mice.

While the study provides compelling evidence that BCL11A represents a limiting factor for OPC differentiation during ageing, the downstream targets and molecular mechanisms through which BCL11A exerts its effects are not directly addressed. As such, the work should be interpreted primarily as identifying a key regulatory node rather than a fully defined molecular pathway.

Overall, this study offers valuable insights into the age-related loss of regenerative capacity in the central nervous system and introduces a computational framework that may be broadly useful for investigating dynamic gene regulation in other biological contexts.

We are grateful to the reviewer for their supportive comments and for highlighting the broader relevance of our computational framework beyond our specific subfield.

Major Points:

(1) MACS mouse anti-A2B5 microbeads are not OPC-specific and may also label astrocyte precursor cells or immature astrocytes. How do the authors justify this caveat? Could some of the claimed "OPCspecific" switch genes in fact be enriched in astrocyte lineage cells?

We thank the reviewer for raising this important point. While anti-A2B5 is a well-established and widely used antibody for isolating OPCs, we nonetheless agree that no technique can isolate a specific cell type with 100% purity, and this also applies to OPC-specific isolation using a validated anti-A2B5 antibody.

To check whether astrocyte contamination could be an issue in determining differential expression, and specifically whether the OPC population was affected by astrocyte contamination, we checked the relative expression and statistical significance of the astrocyte marker Gfap. We refer to our new Supplementary Figure 1 and Supplementary text. This suggests that no difference exists in Gfap levels when comparing OPC, PreOL and OL populations. Therefore, we contend that it is unlikely that the differential expression observed in any cell population is actually due to astrocyte contamination, or that the OPC population is selectively contaminated by astrocytes.

We now included the following in the Supplementary text:

“We did not detect any difference in the astrocyte marker Gfap across these cell-type comparisons, suggesting a similar level of unavoidable astrocytic contamination across groups. Therefore, such contamination is unlikely to confound the interpretation of differential gene expression among OPCs, PreOLs and OLs.”

(2) Overall, Figures 1 and 2 are not very informative in terms of biological insight. The authors should provide more detail in the main figures regarding the enriched gene sets associated with each of the Type 1-4 switch categories. For example, summarizing the top Gene Ontology terms for each switch type would greatly enhance interpretability.

We agree that GO analysis can add further interpretability. We have now prepared a new Supplementary Figure 3A to summarise the significant top GO-term enrichment for switch Types 1– 4, for which gSWITCH-identified patterns are presented in Figure 1C. We also prepared a Supplementary Figure 3B to summarise the top significant GO-term enrichment for the 135 Type 3 switch genes affected in ageing, presented in Figure 2C. Please note that only 8 Type 4 genes overlapped with differentially expressed genes in ageing. Due to this small number, we could not identify any significant GO-term enrichment, and therefore this was not plotted.

(3) A similar issue applies to Figure 3. The authors should explicitly specify the transcription factors in the main figure, particularly the 27 TFs identified through theENCODE/ReMap2 analysis.

Thank you for raising this point. We have now prepared a new Supplementary Table 3, where we list 27 TFs and highlight, with light grey shading, the 5 TFs that overlapped with Type 3 switches.

(4) Have the authors validated Bcl11a expression across different CNS cell types and between young and aged conditions using independent methods such as qPCR, immunofluorescence, or western blotting?

Thank you for this suggestion. We performed qPCR and presented this data in a new Supplementary Figure 4. We found that Bcl11a expression is lower in OLs than in OPCs (Supplementary Figure 4A). We also observed reduced Bcl11a expression in aged OPCs compared with young OPCs (Supplementary Figure 4B). (see also response to Reviewer 1’s recommendations).

(5) Regarding OPC aging, an open question is whether the reduced differentiation capacity of aged OPCs is an intrinsic property of the cells themselves or whether it results from prolonged exposure to an aging environment that induces non-cell-autonomous epigenetic or genetic changes, thereby rendering OPCs less efficient at differentiating. It would be helpful if the authors could expand on this point in the Discussion, with reference to relevant previous studies and experimental evidence.

We thank the reviewer for suggesting this important aspect be discussed. We have now included the following paragraph in the discussion section:

“The extent to which the reduced differentiation capacity of aged OPCs is intrinsically encoded within the cells themselves or induced by prolonged exposure to an aged tissue environment is an interesting question. Based on our previous work, we favour the view that loss of OPC function is primarily determined extrinsically since various manipulations of the aged environment such as heterochronic parabiosis (Ruckh et al., 2012), fasting and calorie restriction mimetics (Neumann et al., 2019), and niche biomechanics (Segel et al., 2019) can all alter the cell-intrinsic state, reverting aged cells to a ‘youthful state’. Significantly, when aged OPCs are transplanted into the neonatal CNS they proliferate and differentiate as if they were neonatal OPCs (Segel et al., 2019). The reversion of aged OPCs to a functional state by changes in their external environment necessarily operates through changes in cell intrinsic function, suggesting that the same intrinsic mechanisms could be targeted directly to restore declining OPC function—for example through epigenetic regulation of differentiation inhibitors (Shen et al., 2008) or overexpression of transcriptional regulators such as c-Myc (Neumann et al., 2021, Dimas et al., 2025).”

(6) Do the authors observe a change in the number or density of OPCs between young and aged mice?

Thank you for asking this important question. In 2002 we reported that there was no difference in the OPCS density between young adult and old adult rats, at least in the deep cerebellar white matter (Sim et al. 2002 - PMID: 11923409). We also refer the reviewer to Figure S1 of another previous study, published in Cell Stem Cell in 2019 (PMID: 31585093). We did not find any difference in OPC number between young and aged brains. Quantification was performed using FACS, where freshly isolated cells were stained with A2B5 (OPC marker), CD11b (microglia marker), and MOG (oligodendrocyte marker). Thus, we do not find any evidence for an age-related decline in OPC densities.

(7) The in vivo characterization of Bcl11a overexpression using the AAV-based approach appears incomplete. Do aged mice overexpressing Bcl11a in Sox10⁺ cells exhibit reduced age-related myelin degeneration under baseline conditions? In the LPC model, do the authors observe differences in lesion size and/or remyelination efficiency?

Again, we thank the reviewer for raising these interesting points. To assess whether Bcl11a overexpression in Sox10+ myelinating oligodendrocytes exhibit less age-related myelin degeneration would, we suspect, require long-term experiments. For this to be the case would require a role for Bcl11a in myelin maintenance – and interesting question but one we feel (and hope the reviewer agrees) is beyond the scope of the current study. We do not see any difference in lesion size (and would not expect the expression of elevated levels of Bcl11a to protect against the membrane-solubilising effects of LPC) but do see changes in remyelination efficiency as shown in Figure 6.

(8) Are the authors presenting gSWITCH for the first time in this manuscript? Given that the gSWITCH framework is novel and central to the study, its conceptual contribution could be emphasized more strongly. A brief comparison with existing trajectory- or pattern-based methods-ideally in the main text around Figure 1-would help readers better appreciate its novelty.

We thank the reviewer for this important suggestion. Yes, gSWITCH is presented for the first time in this manuscript as a new computational framework and web application. We agree that its conceptual contribution should be made clearer in the main text itself, although we explained its concept in detail in ‘Materials and Methods’ and in the supplementary Figure 2 (which was Supplementary Figure 1 in first version of this manuscript).

We now included the following paragraph in the manuscript:

“Existing computational tools such as Monocle (Trapnell et al., 2014), tradeSeq (Van den Berge et al., 2020) and maSigPro (Nueda et al., 2014) are highly valuable for identifying genes with dynamic expression changes across pseudotime or time-course data. gSWITCH addresses a different question. It does not aim to infer trajectories. It works with a user-defined ordered series of biological states or time points and asks a more specific question — does this gene show a statistically supported "switchlike" change in expression as cells move through these states, and if so, what shape does that change take? It combines GLM-based statistical testing with criteria that capture where a gene reaches its highest or lowest expression and whether its expression changes steadily in one direction across the ordered series. To our knowledge, no existing tool combines significance testing with this type of explicit, shape-based classification into discrete, interpretable switch categories. gSWITCH sorts genes into four biologically meaningful patterns, rather than producing only a ranked list of significant genes based on pairwise comparisons between multiple conditions or states. gSWITCH also flags which of these switch genes are transcription factors, making it easier to prioritise candidates for follow-up experiments.

This biologist-friendly tool is freely available as a web application requiring no programming, works with experimental designs containing three or more stages or time points with at least two replicates per stage (no upper limit on either), and can be applied to bulk RNA-seq or to single-cell RNA-seq data aggregated as pseudobulk.”

(9) The evolutionary analysis also appears somewhat disconnected from the rest of the study. Could the authors leverage available public datasets to test whether a similar Bcl11a expression trajectory is observed in human oligodendrocyte lineage cells?

We thank reviewer for mentioning this. We would like to clarify that the evolutionary analysis was included to examine whether Bcl11a sequences across vertebrates, including humans, show evidence of selective constraint, meaning that the sequence has been preserved during evolution because changes in it are likely to be disadvantageous. This analysis was therefore intended to provide broader evolutionary support for the functional importance of Bcl11a, rather than to stand as a separate or disconnected component of the study.

For this analysis, we included Bcl11a DNA and protein sequences from 23 vertebrate species, including humans. We refer the reviewer to the Methods section of this paper, under “dN/dS analysis”, for further details. To provide further clarity regarding the different species used in this study, we have now prepared a new Supplementary Table 4, listing the 23 species together with their DNA and protein sequence accession numbers for Bcl11a.

We also added this sentence in the main text:

“We included twenty-three vertebrate species, including humans (Supplementary Table 4).”

**Recommendations for the authors:**

**Reviewer #1 (Recommendations for the authors):**

Given how central the isolated cells are to the subsequent analysis, the manuscript would be strengthened by a figure showing expression of stage and lineage-specific markers.

Ideally, the authors would provide some sort of orthogonal experimental approach to confirm downregulation of Bcl11a during oligodendrocyte differentiation and loss with age (e.g., IF or RNAScope in conjunction with stage-specific markers in tissue, or western blot in culture).

Thank you again. We have performed these. Please see the Reviewer #1 comment (above).

**Reviewer #2 (Recommendations for the authors):**

(1) Figure 1A: It should be 'anti-O4' instead of 'anti-04'.

This is now corrected. Thank you.

(2) Figure 1C: The authors should specify what the connecting lines indicate (e.g., gene sets or gene modules).

Each coloured line represents one gene and connects its log2 fold-change values across the three oligodendrocyte lineage states: OPC, PreOL and OL. The connecting lines are used to visualize gene-wise patterns of expression change across these cell states. For example, in Type 1, each line shows a pattern in which gene expression increases progressively from OPC to PreOL to OL, with the highest expression change observed in OLs: OL > PreOL > OPC.

We now included the following line in the figure legend:

“Each coloured line represents one gene and connects its log_2_ fold-change values across the three oligodendrocyte lineage states: OPC, PreOL and OL. The connecting lines are used to visualise gene-wise patterns of expression change across these cell states.”

(3) Figure 2C: The authors should specify "DF genes" in the figure legend.

Thank you for pointing this out. This was a typo: it was written as DF, but it should be DE (differentially expressed) genes. We have now corrected this in the figure and spelled out the abbreviation in the legend. Also, DE gene list is accessible through GEO accession: GSE303317. This also mentioned in the figure legend as:

“DE: Differentially expressed. DE gene list is accessible through GEO accession: GSE303317.”

(4) Figure 4C & Figure 5B: the title for the y-axis of the bar graph is confusing. The authors should specify what "#" indicates. Does it represent the counts? What are the thresholding criteria to judge whether an Olig2 cell is MBP-positive or not? It's unclear what the unit is here for the 0-100 scale.

We apologise for the confusion. We used ‘#’, which is a common notation in mathematical and quantitative contexts, to denote counts, so you are correct. We now mentioned in the legend: “The symbol “#” indicates cell count.”

We counted the number of MBP+OLIG2+ cells, divided this by the total number of OLIG2+ cells, and expressed the value as a percentage. For greater clarity, instead of writing #MBP+/#OLIG2+, we have now written #MBP+OLIG2+/#OLIG2+.

Regarding the 0–100 scale, the unit of the Y-axis is percentage, as stated in both figure legends.

The criterion for classifying an OLIG2+ cell as MBP+ was morphological: an OLIG2+ nucleus, shown in white, had to be surrounded by MBP+ staining, shown in red. Cells meeting this criterion were counted as MBP+OLIG2+ cells. Manual counting was performed blinded to sample identity.

(5) Figure 6B: To discriminate from IF staining, the authors should use italic'Plp1' to indicate the RNA in situ results.

Thank you for pointing this out; we have now corrected it.
